# Network pharmacology and experimental evaluation strategies to decipher the underlying pharmacological mechanism of Traditional Chinese Medicine CFF-1 against prostate cancer

**DOI:** 10.18632/aging.205654

**Published:** 2024-03-13

**Authors:** Yong Wei, Mingxia Zhu, Ye Chen, Qianying Ji, Jun Wang, Luming Shen, Xin Yang, Haibin Hu, Xin Zhou, Qingyi Zhu

**Affiliations:** 1 Department of Urology, The Second Affiliated Hospital of Nanjing Medical University, Nanjing 210000, China; 2 Department of Radiation Oncology, The First Affiliated Hospital of Soochow University, Suzhou 215006, China; 3 The First Medicine College, Taizhou Campus of Nanjing University of Traditional Chinese Medicine, Taizhou 225300, China; 4 Department of Urology, Jiangsu Province Hospital of Chinese Medicine, Affiliated Hospital of Nanjing University of Chinese Medicine, Nanjing 210029, China; 5 Department of Oncology, The Affiliated Suqian First People’s Hospital of Nanjing Medical University, Suqian 223812, China; 6 Department of Oncology, The First Affiliated Hospital of Nanjing Medical University, Nanjing 210029, China

**Keywords:** prostate cancer, Traditional Chinese Medicine, CFF-1, network pharmacology, prognosis

## Abstract

Prostate cancer (PCa) is a common malignancy in elderly men. We have applied Traditional Chinese Medicine CFF-1 in clinical treatments for PCa for several years. Here, we aimed to identify the underlying mechanism of CFF-1 on PCa using network pharmacology and experimental validation. Active ingredients, potential targets of CFF-1 were acquired from the public databases. Subsequently, protein-protein interaction (PPI) and the herbs-active ingredients-target network was constructed. A prognostic model for PCa was also constructed based on key targets. *In vitro* experiments using PCa cell lines CWR22Rv1 and PC-3 were carried out to validate the potential mechanism of CFF-1 on PCa. A total of 112 bioactive compounds and 359 key targets were screened from public databases. PPI and herbs-active ingredients-target network analysis determined 12 genes as the main targets of CFF-1 on PCa. Molecular docking studies indicated that the primary active ingredients of CFF-1 possess strong binding affinity to the top five hub targets. DNMT3B, RXRB and HPRT1 were found to be involved in immune regulation of PCa. *In vitro*, CFF-1 was found to inhibit PCa cell proliferation, migration, invasion and induce apoptosis via PI3K-Akt, HIF-1, TNF, EGFR-TKI resistance and PD-1 checkpoint signaling pathways. This study comprehensively elucidates the underlying molecular mechanism of CFF-1 against PCa, offering a strong rationale for clinical application of CFF-1 in PCa treatment.

## INTRODUCTION

Prostate cancer (PCa) ranks as the second deadly malignancy among men worldwide [1]. While early diagnosis, surgery and radiotherapy have enhanced survival rates in patients with PCa, therapeutic options for advanced stages, especially castration-resistant prostate cancer (CRPC), remain constrained [2–4].

Network pharmacology, a bioinformatics method, facilitates the enhancement of drug efficacy, minimization of adverse reactions, and development of new drugs by analyzing complex compositions and disease-related signaling pathways. It has also been instrumental in deciphering the intricate interplay between active components of natural products, diseases, and targets [5, 6].

Traditional Chinese Medicine (TCM), acting on multiple targets rather than a single target, is more systematic in treating corresponding diseases [7]. The efficacy of TCM lies in the synergistic effect of multiple targets and components, regulating diverse biological mechanisms. However, the action mechanism of TCM formulas, with their intricate components, is more complex than that of single medicines [8–10]. Traditional pharmacological studies of TCM, often focused on individual ingredients or medicines, struggle to elucidate the synergistic effects of various chemical constituents.

CFF-1, a TCM obtained from Fusong Xu, a renowned TCM practitioner from Jiangsu Province Hospital of Chinese Medicine, has been employed in clinical settings to treat PCa for several years. According to our previous reports, CFF-1 suppressed cell growth and promoted apoptosis through EGFR-related pathways in PCa [11]. It is also reported to counteract PCa through suppressing PD-1/PD-L1 checkpoint signaling via EGFR-related pathways [12]. Nevertheless, the comprehensive mechanism of CFF-1 in treating PCa has yet to be fully elucidated using robust methodologies. Network pharmacology, merging the advantages of TCM with the most advanced medical technology, has become a favorable approach for TCM research [13, 14].

We utilized a comprehensive network pharmacology and molecular docking approach to investigate the bioactives of CFF-1, predict their effective targets, and understand the underlying molecular mechanisms in PCa. A prognostic model based on key targets was also constructed. Finally, we verified the predicted results in PCa cell lines.

## RESULTS

### Active compounds in CFF-1 and target screening of CFF-1 on PCa

**Figure 1:**
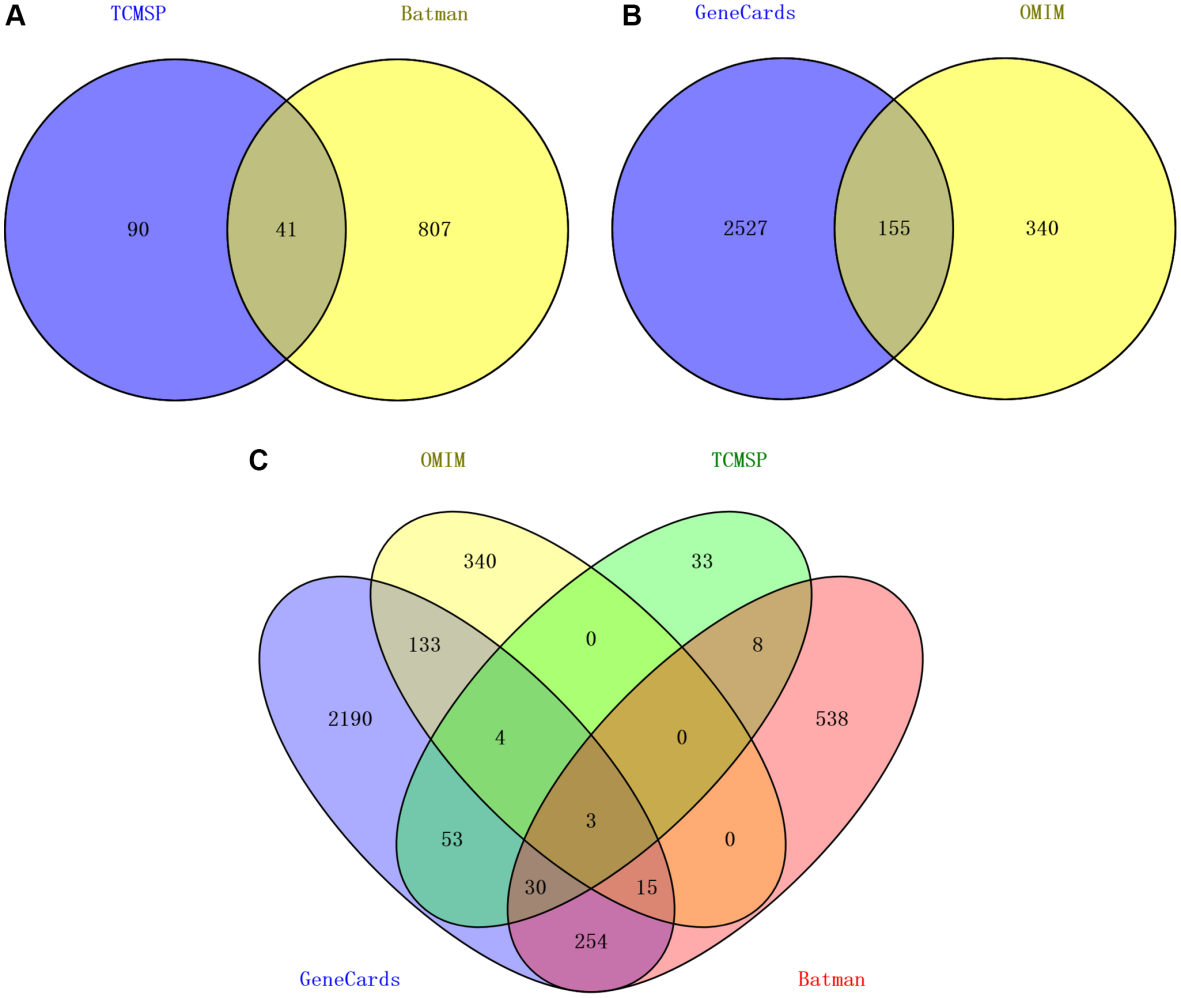
**Target screening of CFF-1 on PCa.** (**A**) Venn diagrams showing CFF-1 targets obtained from TCMSP and BATMAN-TCM. (**B**) Targets related to PCa acquired from Genecards and OMIM. (**C**) The intersection of targets for both CFF-1 and PCa.

Initially, 78 active compounds in CFF-1, each with OB ≥30% and DL ≥0.1 in CFF-1, were collected from TCMSP platform. Subsequently, 70 active compounds with *p* < 0.05, score >20 were obtained from BATMAN-TCM platform. After integrating these findings, 112 active compounds were selected for further study (Table 1). The targets for the candidate compounds in CFF-1 were explored from TCMSP and BATMAN-TCM, identifying 131 and 848 putative targets, respectively. There were 41 overlapping targets among the two sets. Ultimately, 938 targets for CFF-1 active components were acquired by integrating the overlapping targets (Figure 1A). Additionally, 2682 target genes related to PCa were acquired from the Genecards by setting the correlation score >20 and 495 target genes were acquired from OMIM databases. A total of 3022 target genes acquired from the two sets after eliminating the overlaps (Figure 1B). Nine hundred and thirty-eight targets for drug active components and 3022 targets for PCa were screened out by Perl language program and R language software. In total, 359 overlapping target genes were recognized as key targets related to both CFF-1 and PCa for further analyses (Figure 1C).

**Table 1:** Active compounds in CFF-1.

**Number**	**Active compounds**	**Number**	**Active compounds**	**Number**	**Active compounds**
1	Isoliquiritigenin	2	DFV	3	baicalein
4	3′-Methoxydaidzein	5	beta-sitosterol	6	sitosterol
7	(Z)-1-(2,4-dihydroxyphenyl)-3-(4-hydroxyphenyl)prop-2-en-1-one	8	(2R)-7-hydroxy-2-(4-hydroxyphenyl)chroman-4-one	9	1H-Cycloprop(e)azulen-7-ol, decahydro-1,1,7-trimethyl-4-methylene-,(1aR-(1aalpha,4aalpha,7beta,7abeta,7balpha))
10	4′,5-Dihydroxyflavone	11	2-Acridinecarboxylic acid	12	(Z)-nonadec-6-enoic acid
13	Azetidine-2-Carboxylic Acid	14	Aspartic Acid	15	Digitalis Glycoside
16	Homoserine	17	Mannose	18	EIC
19	Aeginetic acid	20	jioglutin D	21	METHYL PALMITOLEATE
22	Stigmasterol	23	Uridine	24	DMEP
25	1,2-Dibenzoylethane	26	WLN: RVO2R	27	(−)-taxifolin
28	ELD	29	diosgenin	30	()-alpha-Longipinene
31	(+)-catechin	32	(−)-Caryophyllene oxide	33	(−)-alpha-cedrene
34	DBP	35	ent-Epicatechin	36	alpha-Longipinene
37	DIBP	38	()-Aromadendrene	39	beta-Cubebene
40	(−)-Epoxycaryophyllene	41	oleic acid	42	Hepanal
43	58870_FLUKA	44	()-alpha-Funebrene	45	phytol
46	8-Deoxy-14-Dehydro-Aconosine	47	1,2-Benzenedicarboxylicacid, mono(2-ethyl) hexylester	48	(−)-Alloaromadendrene
49	Procurcumenol	50	Tetradecanal	51	Cinnamaldehyde
52	5-Cinnamoyl-9-O-Acetylphototaxicin I	53	Anethole	54	Protocatechuic Acid
55	Coumarinic Acid	56	Gamma-Sitosterol	57	Camphor
58	Melilotocarpan A	59	Farnesol	60	Nerolidol
61	Trans-Cinnamic Acid	62	Styrene	63	11,14-eicosadienoic acid
64	Delphin_qt	65	Deltoin	66	Deoxyandrographolide
67	Karanjin	68	Talatisamine	69	Benzoylaconine
70	Aconitine	71	Delgrandine	72	Aconine
73	14-Deoxy-11,12-Didehydroandrographolide	74	Deltaline	75	Delavaconitine
76	Deltamine	77	Carmichaeline	78	Delsoline
79	Salsolinol	80	Crassicauline A	81	Delphamine
82	Bullatine B	83	Benzoylhypaconine	84	Bullatine C
85	Coryneine	86	Vilmorrianine C	87	3-Acetylaconitine
88	Deoxyaconitine	89	Delphatine	90	M-Aminophenol
91	Karakoline	92	Hypaconitine	93	Talatizamine
94	Neojiangyouaconitine	95	Ignavine	96	Ortho-Aminophenol
97	Para-Aminophenol	98	Neokadsuranic Acid B	99	Benzoylmesaconine
100	taxifolin	101	Delbrusine	102	Mescaline
103	Higenamine	104	Carnosifloside I	105	Mesaconitine
106	Isotalatizidine	107	Neoline	108	P-Aminophenol
109	Delcorine	110	Delbrusine	111	Karacoline
112	Delbruline				

### Compound-target network and analysis

**Figure 2:**
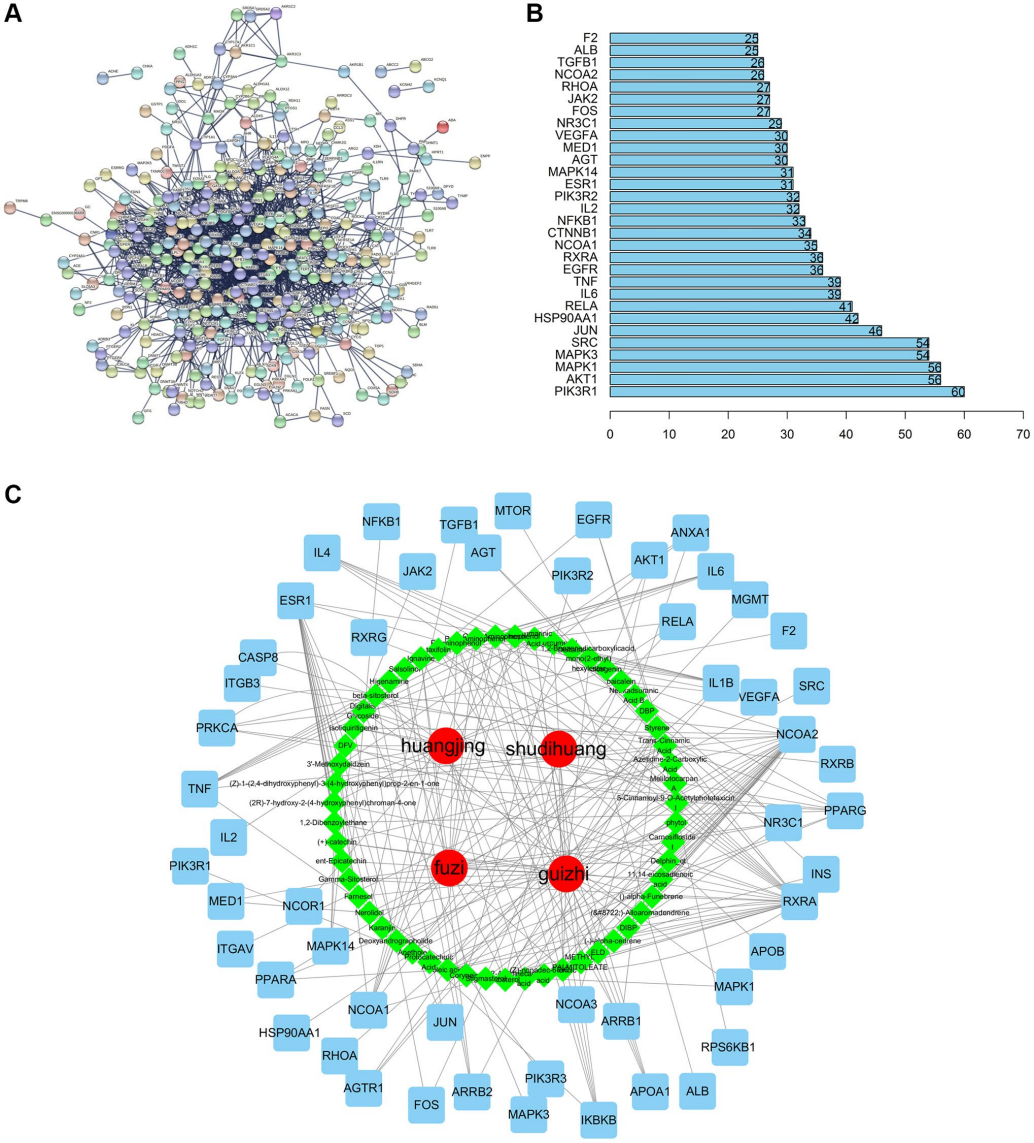
**Compound-target network and analysis.** (**A**) Key targets PPI network of CFF-1 on PCa. (**B**) Bar graph showing the top 30 targets with high degree. (**C**) The herbs-active ingredients-target network of CFF-1 on PCa.

Upon entering the above 359 key targets into STRING, we obtained a key targets PPI network of CFF-1 on PCa. Subsequent analysis of this PPI network focused on “degree” was applied to select the target in the core position (Figure 2A). The top 30 target genes with high degree were shown in Figure 2B. The 53 core targets were further screened by setting interaction score ≥0.9 and degree ≥20. Cytoscape software was applied to construct the herbs-active ingredients-target network, containing 112 nodes (55 for candidate active ingredients and 53 for core targets) and 253 edges. In the network, the nodes with more edges might play essential roles in the pharmacological processes, and 12 nodes (edge ≥5) were determined as the main targets of CFF-1 on PCa for further analysis, including NCOA2, RXRA, ESR1, NCOA1, PPARG, IL1B, TNF, IKBKB, NR3C1, IL4, IL6 and PRKCA (Figure 2C).

### Molecular docking verification

**Figure 3:**
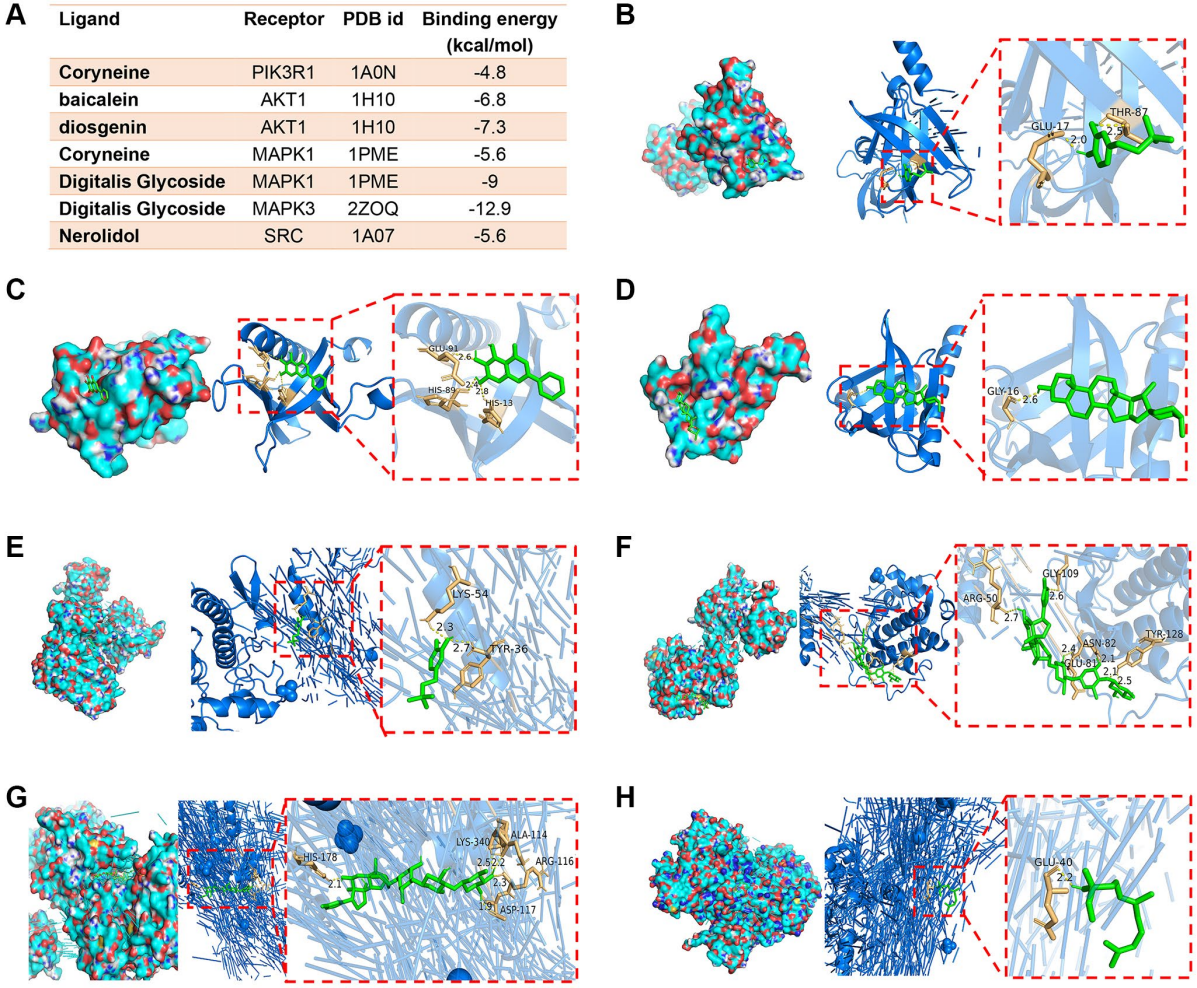
**Representative images of molecular docking.** (**A**) The results of ligand-receptor binding energy values. (**B**) Coryneine-PIK3R1. (**C**) Baicalein-AKT1. (**D**) Diosgenin-AKT1. (**E**) Coryneine-MAPK1. (**F**) Digitalis glycoside-MAPK1. (**G**) Digitalis glycoside-MAPK3. (**H**) Nerolidol-SRC.

As previously mentioned, PIK3R1, AKT1, MAPK1, MAPK3 and SRC were the top five hub targets in PPI network. These targets were then docked with their respective active ingredients. The ligand-receptor binding energy values, indicative of binding stability, were presented in Figure 3A. Generally, a binding energy lower than −5 kcal/mol is considered indicative of stable binding. Notably, coryneine establishes two hydrogen bonds with GLU-17 and THR-18 in PIK3R1, while baicalein forms three hydrogen bonds with GLU-91, HIS-89 and HIS-13 in AKT1. Diosgenin forms one hydrogen bond with GLY-16 in AKT1. Coryneine and digitalis glycoside bound to MAPK1 with binding energy values of −5.6 kcal/mol and −9 kcal/mol, respectively. Among these, the binding affinity of digitalis glycoside to MAPK3 was the strongest, with a value of −12.9 kcal/mol. Additionally, nerolidol forms one hydrogen bond with GLU40 in SRC (Figure 3B–3H).

### Enrichment analysis

**Figure 4:**
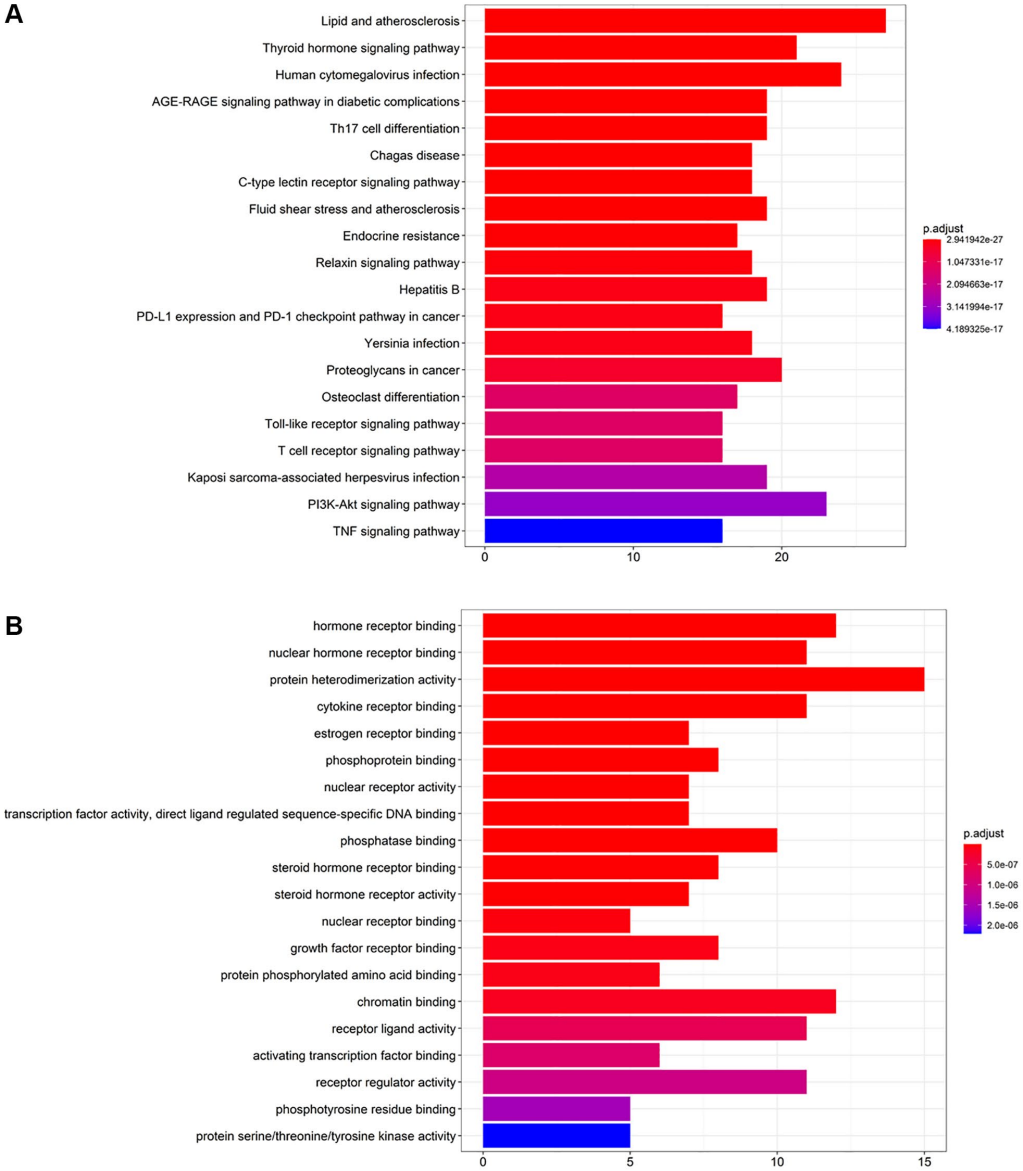
**KEGG pathway enrichment and GO biological process analysis of key targets of CFF-1 on PCa.** (**A**) The top 20 enriched pathways of KEGG pathway enrichment analysis. (**B**) The top 20 enriched terms of GO biological process analysis.

To ascertain the involved pathways of CFF-1 on PCa, we carried out KEGG pathway analysis of 359 key targets. A total of 172 involved pathways were identified (Supplementary Table 1). Figure 4A displayed the top 20 enriched pathways, suggesting that the anti-cancer effect of CFF-1 on PCa likely results from a relatively complex and multi-pathway synergistic effect.

To further explore the biological role of involved targets of CFF-1 against PCa, the GO process analysis of 359 key targets was performed. A total of 238 GO terms were found (Supplementary Table 2). Figure 4B displayed the top 20 enriched terms related to PCa, suggesting that CFF-1 may exhibit its therapeutic effects through the above biological processes.

### Prognostic model construction based on key targets

**Figure 5:**
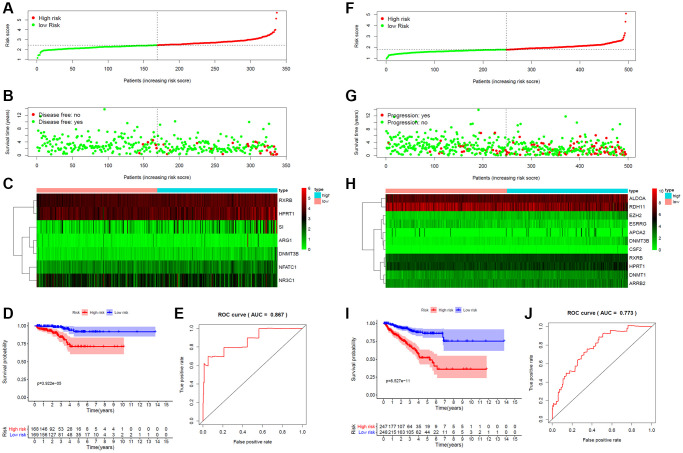
**Risk score for target gene signature and outcome in PCa patients.** (**A**) Risk score of a 7-gene signature for predicting DFS. (**B**) Disease status and duration of cases. (**C**) Heatmap of the 7 gene expression in PCa patients. (**D**) Kaplan-Meier curve for DFS in the low- and high-risk groups. (**E**) ROC curve for the DFS prediction of risk score model. (**F**) Risk score of a 11-gene signature for predicting PFS. (**G**) Progression status and duration of cases. (**H**) Heatmap of the 11 gene expression in PCa patients. (**I**) Kaplan-Meier curve for PFS in the low- and high-risk groups. (**J**) ROC curve for the PFS prediction of risk score model.

A total of 359 key target genes were subjected to LASSO regression analysis to identify genes for construction of risk score model for PCa patients in the TCGA database. The risk score for predicting DFS of each PCa patient was calculated as follows: Risk score = NFATC1 × 0.234 + ARG1 × 0.001 + DNMT3B × 0.315 − NR3C1 × 0.025 + RXRB × 0.105 + HPRT1 × 0.033 + SI × 0.037. A 7-gene signature was constructed. Patients were classified into high- and low-risk groups based on the median risk score (Figure 5A). A risk curve and a scatter plot were applied to show the risk score and the survival status of each PCa patient, respectively. Most recurrent cases were distributed in the high-risk group (Figure 5B). The expression profile of candidate genes indicated that NFATC1, ARG1, DNMT3B, NR3C1, RXRB, HPRT1 and SI were highly expressed in the high-risk group, except for NR3C1 (Figure 5C). PCa patients in the high-risk group had a significantly worse DFS than those in the low-risk group. AUC of ROC curve for the DFS prediction of risk score model was 0.867 (Figure 5D, 5E).

The risk score for predicting PFS was calculated as follows: Risk score =ALDOA × 0.0003 + DNMT3B × 0.222 + CSF2 × 0.099 + DNMT1 × 0.031 + EZH2 × 0.054 + ARRB2 × 0.019 − ESRRG × 0.025 + APOA2 × 0.007 + RXRB × 0.076 + HPRT1 × 0.021 − RDH11 × 0.0001. A 11-gene signature was constructed (Figure 5F). Progression patients were mainly distributed in the high-risk group (Figure 5G). The expression profile showed that ALDOA, DNMT3B, CSF2, DNMT1, EZH2, ARRB2, APOA2, RXRB and HPRT1 were highly expressed in the high-risk group, except for ESRRG and RDH11 (Figure 5H). Kaplan–Meier curves showed that PFS was significantly worse in high-risk patients than low-risk patients. AUC of ROC curve for the PFS prediction was 0.773 (Figure 5I, 5J).

For OS, though few patients reached the endpoint (10 of 495 patients), the risk score model was also constructed. The risk score for predicting OS was calculated as follows: Risk score = AR × 0.048 + AGTR1 × 0.062 + PPARD × 0.427 + PHB × 0.090 + RPS6KB1 × 0.620 + FADD × 0.821 − DNMT1 × 0.667 +AKR1C3 × 0.075. An 8-gene signature was constructed (Supplementary Figure 1A). More dead cases were found in the high-risk group (Supplementary Figure 1B). The heat map suggested that AR, AGTR1, PPARD, PHB, RPS6KB1, FADD, DNMT1 and AKR1C3 were overexpressed in the high-risk group, except for DNMT1 (Supplementary Figure 1C). Patients in the high-risk group had significantly shorter OS compared with those in the low-risk group. AUC of ROC curve for the OS prediction was 0.992 (Supplementary Figure 1D, 1E). Taken together, these gene signatures might effectively predict the prognosis of PCa and may act as potential targets for PCa therapy.

We then explored possible associations of risk scores with ESTIMATE score using the ESTIMATE algorithm. Using the CIBERSORT algorithm, the correlation between risk score and the infiltration of 22 immune cell subtypes were assessed. The risk score for predicting OS of PCa patient was negatively associated with stromal score, immune score, and ESTIMATE score, while positively with the infiltration of mast cells resting (Supplementary Figure 2A). The risk score for predicting DFS was negatively related to the infiltration of plasma cells, while positively Tregs and macrophages M2 (Supplementary Figure 2B). The risk score for predicting PFS was positively related to stromal score, immune score, ESTIMATE score, the infiltration of Tregs macrophages M1 and macrophages M2, while negatively with plasma cells and mast cells resting (Supplementary Figure 2C).

DNMT3B, RXRB and HPRT1 were the common target genes affecting both PFS and DFS in PCa patients. GO functional annotations and KEGG pathways enrichment were applied to evaluate the biological significance of DNMT3B, RXRB and HPRT1 in PCa. The results indicated that DNMT3B, RXRB and HPRT1 were widely involved in immune regulation (Supplementary Figure 3).

### CFF-1 inhibited PCa cells proliferation and induced apoptosis

**Figure 6:**
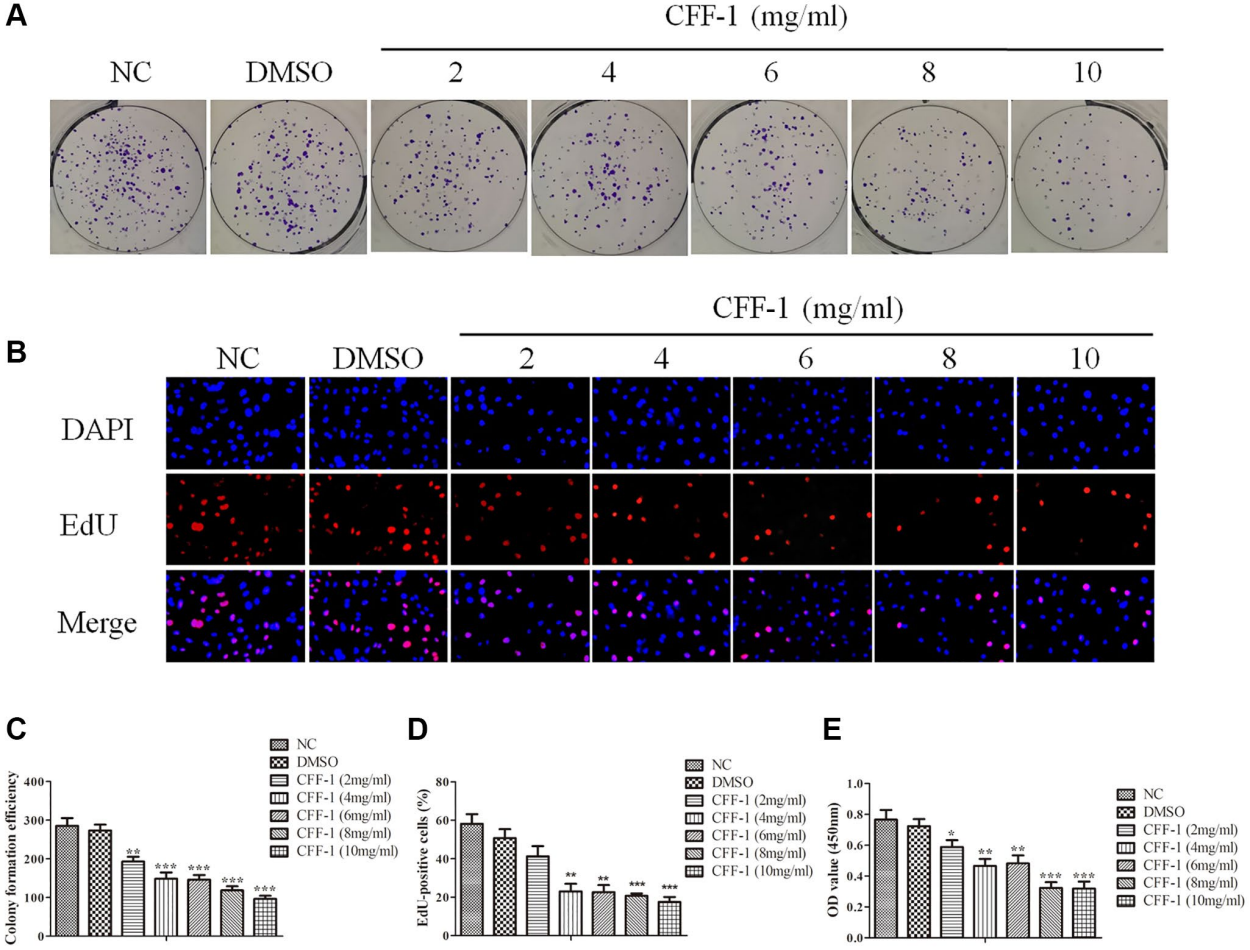
**CFF-1 inhibited PCa cells proliferation.** (**A**) The colony formation efficiency of PC-3 cell lines with varying concentrations of CFF-1 (mg/mL). (**B**) The EdU assay of proliferation active cells after 24 hours of CFF-1 treatment. (**C**) Quantification of the colony formation efficiency with bar graph. (**D**) Quantitative results of the EdU assay with bar graph. (**E**) The cell viability was determined by CCK8 assay after CFF-1 administration. ^*^p < 0.05, ^**^p < 0.01, ^***^p < 0.001.

**Figure 7:**
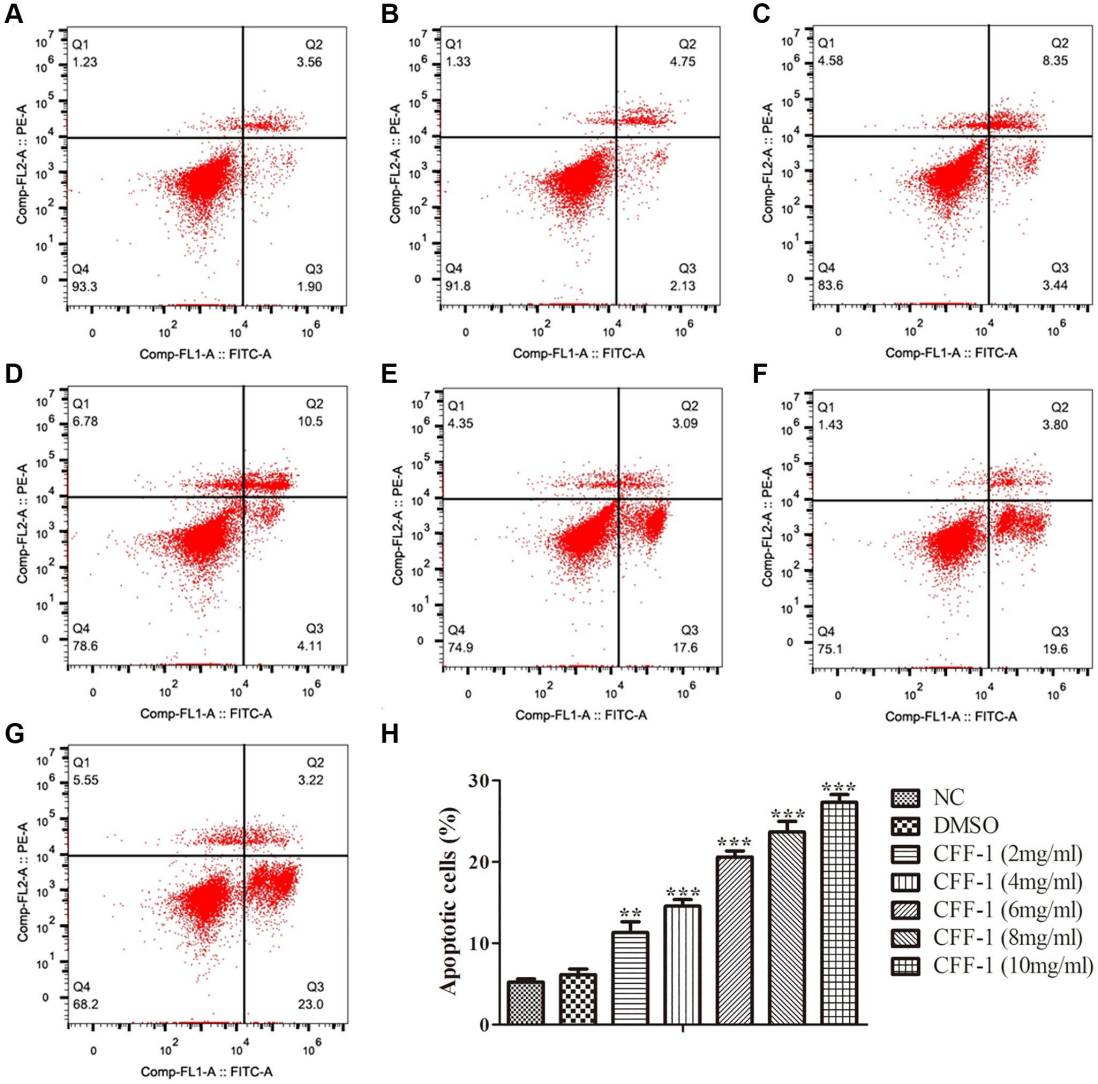
**Apoptosis analysis of CFF-1-treated PC-3 cells by flow cytometry after 24 hours of treatment.** Each panel corresponds to a different treatment condition: (**A**) NC, (**B**) DMSO, and (**C**–**G**) increasing concentrations of CFF-1 at 2, 4, 6, 8, and 10 mg/ml, respectively. (**H**) Quantitative analysis of the percentage of apoptotic cells across different treatment conditions. ^**^p < 0.01, ^***^p < 0.001.

To evaluate the effect of CFF-1 on PCa as postulated from network pharmacology analysis, clonogenic assay, CCK8 and Edu assays were conducted in PC-3 cell line treated with different concentrations of CFF-1 (0, 2, 4, 6, 8 and 10 mg/ml). The cell colony formation efficiency of PC-3 cells was decreased dose dependently compared to the negative control (Figure 6A, 6C). Furthermore, a dose-dependent reduction in proliferation active cells was observed after 24 hours of CFF-1 treatment in the EdU assay (Figure 6B, 6D). The CCK8 assay confirmed a dose-dependent decrease in PCa cell viability (Figure 6E). In addition, CFF-1 treatment increased the percentage of apoptotic PC-3 cells dose dependently (Figure 7A–7H).

### CFF-1 inhibited PCa cells migration and invasion

**Figure 8:**
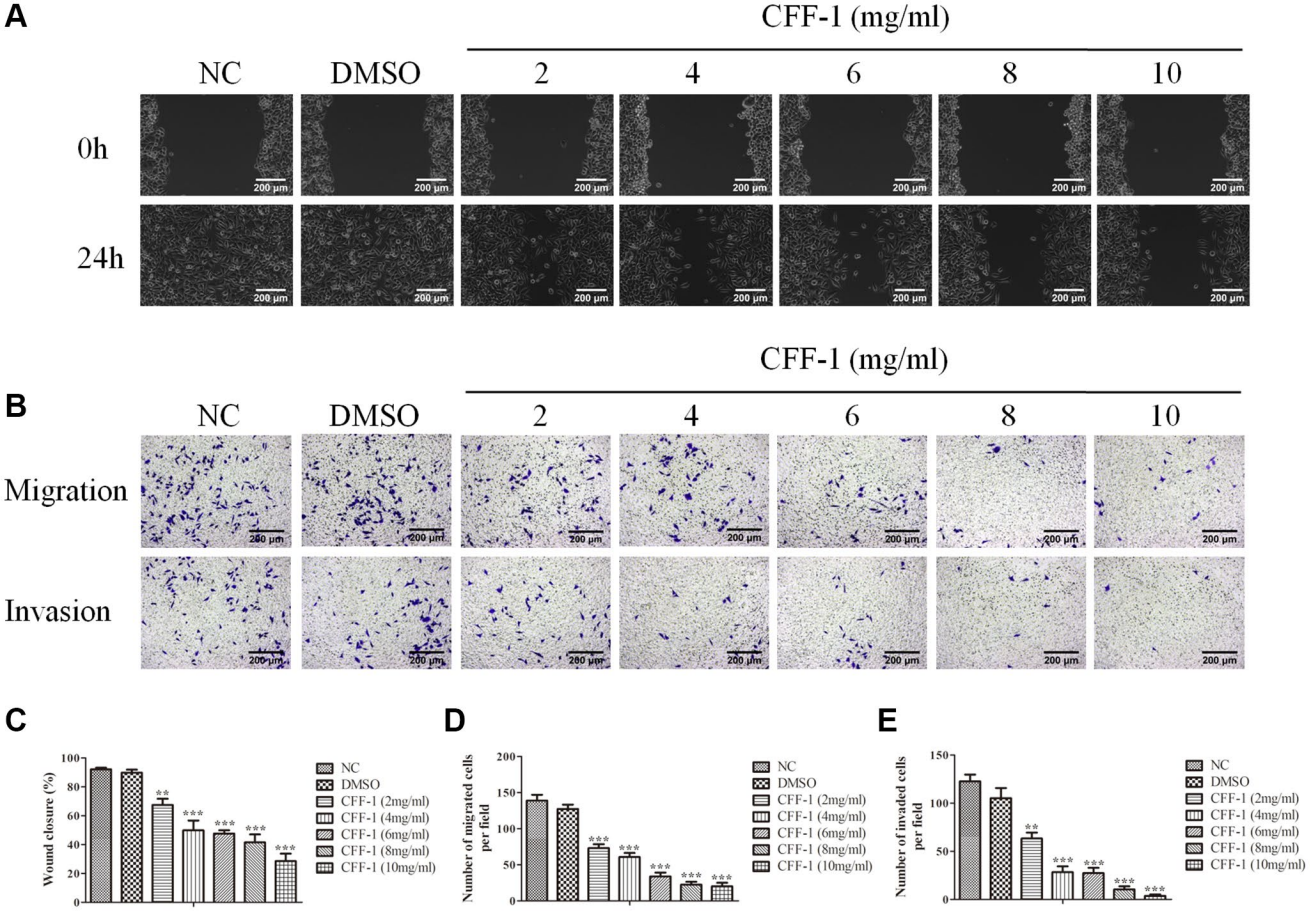
**CFF-1 inhibited PCa cells migration and invasion.** (**A**) Wound healing abilities of PC-3 cells in the presence of NC, DMSO and varying concentrations of CFF-1, with images captured at 0 and 24 hours post-wounding. (**B**) Representative images showing transwell migration and invasion assay of PC-3 cells treated with NC, DMSO and varying concentrations of CFF-1. (**C**) Quantitative analysis of the wound healing assay with different treatment conditions. (**D**) Quantitative analysis of the transwell migration assay. (**E**) Quantitative analysis of the transwell invasion assay. ^**^p < 0.01, ^***^p < 0.001.

Subsequently, wound healing and transwell experiments were conducted to explore the migration and invasion abilities of PC-3 cells treated with CFF-1. We found that fewer cells migrated to the scratch site after 24 hours of CFF-1 treatment dose dependently (Figure 8A, 8C). Furthermore, the migration and invasion ability of PC-3 cells were significantly inhibited after treatment with increasing CFF-1 (Figure 8B, 8D, 8E).

### CFF-1 attenuated proliferation pathways in PCa cells

**Figure 9:**
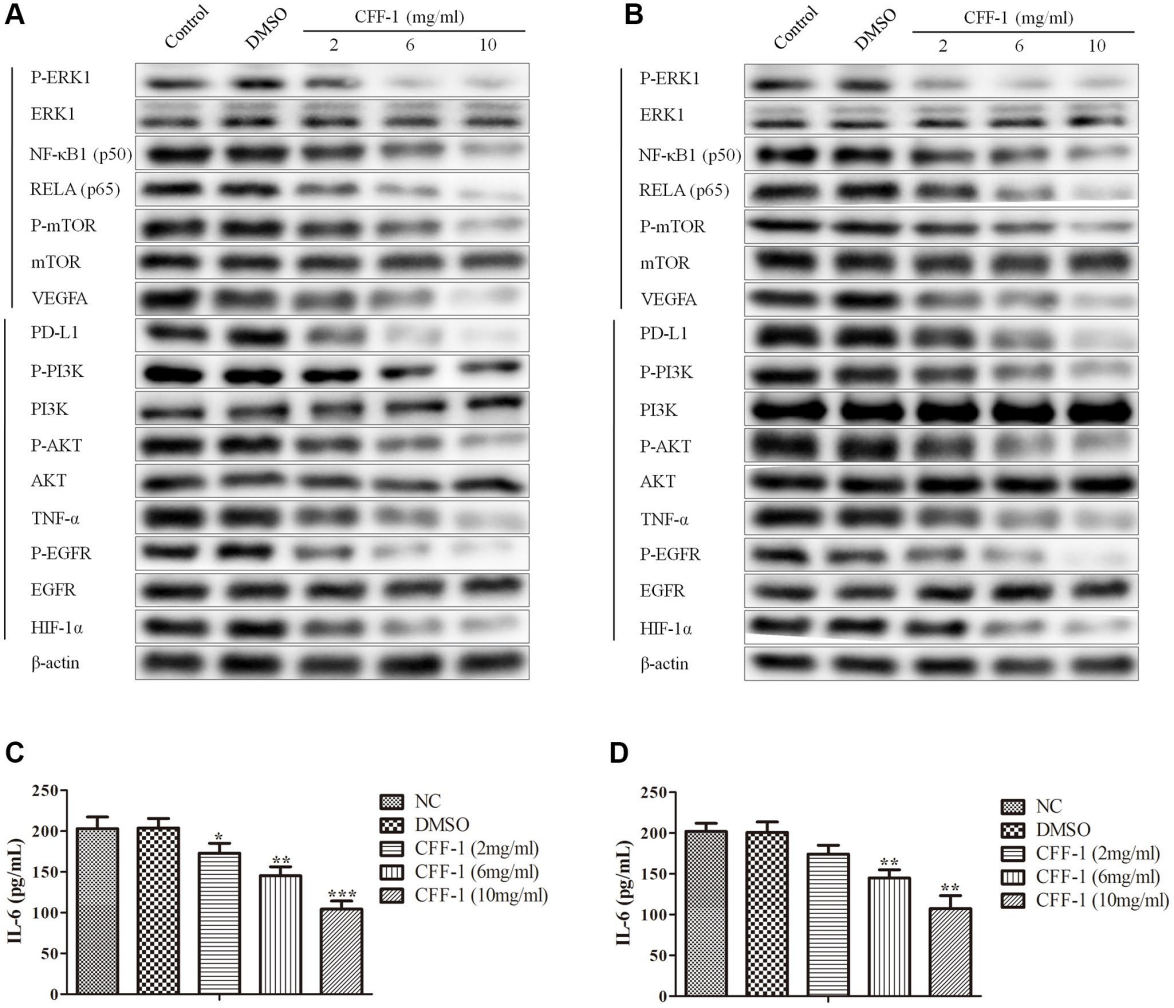
**The relative expressions of related proteins with CFF-1 treatment on PCa cells.** (**A**) The relative expressions of related proteins after 24 hours of CFF-1 treatment on PC-3 cells. (**B**) The relative expressions of related proteins after 24 hours of CFF-1 treatment on 22RV1 cells. (**C**) The secretory levels IL-6 after 48 hours of CFF-1 treatment on PC-3 cells. (**D**) The secretory levels IL-6 after 48 hours of CFF-1 treatment on 22RV1 cells.

According to network pharmacology analysis, the PI3K-Akt, HIF-1, TNF, EGFR-TKI resistance and PD-1 checkpoint pathway might play a crucial role in regulating PCa cell proliferation and survival by CFF-1. Then, we evaluated the expressions level of the common key targets of the five pathways. Pretreatment of CWR22Rv1 and PC3 cells with CFF-1 (2, 6 and 10 mg/ml) resulted in apparent repression of P-ERK1, NFκB1, RELA, P-mTOR, VEGFA, PD-L1, P-PI3K, P-AKT, TNF-α, P-EGFR and HIF-1α in dose dependently (Figure 9A, 9B). ELISA assays indicated that the secretory IL-6 of CWR22Rv1 and PC3 cells was blocked by CFF-1 dose dependently (Figure 9C, 9D). These findings suggested that the above five pathways might be crucial for CFF-1 anti-cancer effect in PCa.

## DISCUSSION

In our previous study, the administration of CFF-1 in patients with metastatic castration-resistant prostate cancer (mCRPC) resulted in a significant reduction in Prostate-Specific Antigen (PSA) levels. This reduction is an important marker of CFF-1’s therapeutic efficacy in slowing prostate cancer progression. Beyond this quantifiable impact on PSA levels, the study also recorded improvements in clinical symptoms associated with mCRPC. Patients undergoing CFF-1 treatment experienced alleviated symptoms, reflecting an improvement in their quality of life. Additionally, a notable decrease in fatigue was observed among patients receiving CFF-1. Our collective research has also shown that CFF-1 exerts potent anti-tumor immunity, effectively hindering tumor growth and metastasis in prostate cancer via the EGFR/JAK1/STAT3 pathway, subsequently inhibiting PD-1/PD-L1 checkpoint signaling. Additionally, CFF-1 promotes cell growth inhibition, autophagy, and apoptosis by targeting and inhibiting EGFR-related pathways in PCa [11–12]. Network pharmacology combined with molecular docking have been used as a valuable tool to explore the complex mechanisms of TCM [5]. In this study, the role of CFF-1 was comprehensively elucidated through a multifaceted approach encompassing network pharmacology, bioinformatics, and *in vitro* validation. This approach, transcending traditional single-target strategies, aligns with the emerging paradigm shift in oncology towards multi-targeted therapies.

In our study, 112 bioactives and 359 key targets were screened, thus unveiling an extensive molecular framework for potential intervention in PCa. The herbs-active ingredients-target network demonstrated that NCOA2, RXRA, ESR1, NCOA1, PPARG, IL1B, TNF, IKBKB, NR3C1, IL4, IL6 and PRKCA could serve as main targets for CFF-1 on PCa. According to molecular docking results, the primary active ingredients exhibited a robust binding affinity to the top five hub targets, primarily through the formation of hydrogen bonds. This data not only enriches the current understanding of PCa but also opens new avenues for targeted therapy.

The prognostic model we developed, anchored on these key targets, could be instrumental in tailoring personalized treatment regimens, a cornerstone of contemporary oncology. According to KEGG analysis, CFF-1 could have anti-cancer effects against PCa by regulating cancer cell proliferation and survival through PI3K-Akt, HIF-1, TNF, EGFR-TKI resistance and PD-1 checkpoint signaling pathways. This multi-pathway approach underlines the complexity and synergy of TCM in treating diseases. We carried out a series of biological function assays in diverse PCa cell lines to validate the anti-PCa ability of CFF-1. The observed inhibition of cell proliferation, migration, invasion, and induction of apoptosis in PCa cell lines are congruent with our network pharmacology predictions. This is also in line with our previous studies of PCa [11, 12].

The PI3K/Akt signaling pathway, a vital intracellular pathway, involved in tumor progression of various malignant tumors [15]. This pathway emerges as a central oncogenic axis in PCa, orchestrating a spectrum of cellular activities including proliferation, apoptosis, cell cycling, metastasis, and drug resistance [16–20]. The modulation of PI3K/Akt by CFF-1, as evidenced in our findings, could thus represent a significant stride in targeting these fundamental oncological processes. Further, our study sheds light on the role of tumor necrosis factor (TNF), predominantly produced by activated macrophages and T lymphocytes. TNF-α, the macrophage-derived variant, is known to bind to its receptor TNFR1, triggering pathways that not only elicit inflammatory responses but also induce cellular death [21, 22]. Notably, in the context of PCa, TNF-α has been implicated in promoting cell migration via the upregulation of CCR7, particularly in cases of lymph node metastasis [23]. This finding underscores the potential of targeting TNF-α as a means to impede the metastatic progression of PCa. The HIF-1 signaling pathway, another focus of our study, is critically involved in tumor pathogenesis. Activated under hypoxic conditions, this pathway is linked to tumor development, progression, and resistance to therapy, especially in PCa [24–28]. Overexpression of HIF-1α, a key component of this pathway, has been associated with poor prognoses in PCa patients [29]. Consequently, inhibiting HIF-1, as indicated by our study’s results, may provide therapeutic benefits, corroborating findings from previous genetic and pharmacological studies [30–32]. IL-6, a major inflammatory factor within the tumor microenvironment, is intricately involved in tumor progression, immune modulation, and inflammatory responses [33]. Its overexpression in PCa is linked to tumor proliferation, invasion, metastasis, and the development of castration resistance [34–36]. Our research identifies IL-6 as a primary target of CFF-1, emphasizing its potential role in mitigating these malign processes. Additionally, our study delves into the realm of immune checkpoint inhibitors (ICIs), particularly targeting the PD-1/PD-L1 axis, a novel therapeutic avenue in PCa management, especially in metastatic CRPC [37]. The overexpression of PD-L1 in PCa has been correlated with poor clinical outcomes [38], highlighting the potential of ICIs in combination with other treatments as a breakthrough in PCa therapy. EGFR, a transmembrane receptor tyrosine kinase, is known for its role in activating a range of signaling pathways that contribute to tumorigenesis and progression in PCa [39–40]. Our verification of protein expression levels of key targets, including P-ERK1, NFκB1, RELA, P-mTOR, VEGFA, PD-L1, P-PI3K, P-AKT, TNF-α, P-EGFR, HIF-1α, and IL-6 in CWR22Rv1 and PC3 cell lines, reveals that CFF-1 can markedly downregulate these proteins, demonstrating its multifaceted therapeutic efficacy. This multi-target approach of CFF-1 suggests its potential as a comprehensive treatment option, addressing various biological processes and pathways implicated in PCa.

## CONCLUSION

We demonstrated the underlying mechanism of CFF-1 against PCa based on network pharmacology and experimental evaluation. The findings may provide a strong rationale for clinical application of CFF-1 in PCa treatment.

## METHODS

### Screening for active ingredients of CFF-1

All ingredients of four main compounds (guizhi, fuzi, shudihuang, huangjing) in CFF-1 were acquired from TCM Systems Pharmacology Database and Analysis Platform (TCMSP, http://tcmspw.com/tcmsp.php) and a Bioinformation Analysis Tool for Molecular mechanism of Traditional Chinese Medicine (BATMAN-TCM, http://bionet.ncpsb.org/batman-tcm/). The criteria to screen for active ingredients were set as the oral bioavailability (OB) ≥30% with the drug similarity (DL) ≥0.1, and *p* < 0.05 with score >20, respectively.

### Target prediction related to CFF-1 and PCa

The potential targets of active ingredients in CFF-1 were obtained from TCMSP and BATMAN-TCM. Targets related to PCa were acquired from Gene Cards database (https://www.genecards.org/) and Online Mendelian Inheritance in Man database (OMIM, http://www.omim.org/) with a keyword “prostate cancer”. The UniProt database (https://www.uniprot.org/) was applied to standardize all target information, and genes without UniProt ID from human samples were eliminated. Perl software was applied to screen intersecting key targets related to both CFF-1 and PCa.

### Protein–protein interaction analysis and network construction

STRING database (https://string-db.org/) is used to construct the relationship between multiple proteins. We put the key targets of CFF-1 and PCa into the STRING database Version 11.0, selecting the Homo sapiens for the species to perform the PPI network with Cytoscape software Version 3.9.1. Targets with interaction score ≥0.9 and degree ≥20 were regarded as core targets. In order to study the relationship among CFF-1, the core targets and PCa, main herbs, active ingredients and core targets were imported into the Cytoscape software to construct the herbs-active ingredients-target network.

### Molecular docking

Molecular docking was carried out to evaluate the interaction between the top five targets, which exhibited high degree, and their respective active ingredients. Using the PubChem database, we acquired the 3D molecule structures of ingredients. The protein crystal structures of the targets were retrieved from the RCSB PDB database. The PyMOL software facilitated the removal of water molecules and the separation of ligands. For the conversion of small molecules and target proteins into pdbqt format, AutoDock Tools 1.5.6 was utilized. Molecular docking was performed using AutoDock Vina software. Visualization and analysis of the docking results were achieved using the PyMOL tool.

### Enrichment analyses

The Gene Ontology (GO) analysis and Kyoto Encyclopedia of Genes and Genomes (KEGG) pathway enrichment were carried out through gene set enrichment analysis (GSEA) in R Version 3.7.0. Biological significance was defined as *p* < 0.05, and *Q* < 0.05.

### Prognostic model construction based on key targets

RNA sequencing datasets and survival information for PCa were obtained from TCGA data portal. LASSO Cox regression was performed to screen genes with prognostic values from the key targets using R package “glmnet”. A risk score formula for predicting prognosis was established as follows: risk score = expgene1 × βgene1 + expgene2 × βgene2 + … expgenen × βgene. Kaplan–Meier method was used to assess the prognostic significance of the risk score model on PCa by the R package “survival”. Receiver operating characteristic (ROC) curve analysis was performed to explore the predictive power of risk score using the R package “timeROC”.

We applied the CIBERSORT algorithm to assess the correlation between risk score and the infiltration of 22 immune cell subtypes [41]. Based on the ESTIMATE algorithm, we evaluated the relationship between risk score and immune score, stromal score and ESTIMATE score [42].

### Cell culture and drug preparation

The human PCa cell lines CWR22Rv1 and PC-3 were cultured in RPMI-1640 medium, enriched with 10% fetal calf serum (Gibco, Waltham, MA, USA) and maintained in a humidified atmosphere with 5% CO_2_ at 37°C. CFF-1, a TCM herbal mixture, has been detailed in our previously published study [11]. A final concentration of 0, 2, 4, 6, 8 and 10 mg/mL of CFF-1 was used to treat cells.

### Cell viability assay

PCa cell viability was detected by Cell Counting Kit-8 (CCK8) assay kit (Beyotime, Guangzhou, China) based on the manufacturer’s instructions. PC-3 cells were seeded into 96-well plates at a density of 2 × 10^3^ cells/well. Each well was measured at 450 nm for its absorbance.

### EdU assay

In order to further evaluate cell proliferation ability, PC-3 cells were measured using the BeyoClick™ EdU-555 assay according to the manufacturer’s protocol (Beyotime, Guangzhou, China). The signals were measured by fluorescence microscopy Olympus CKX53.

### Clonogenic assay

PC-3 cells were transplanted into 6-well plates with a density of 2 × 10^3^ cells/well and then the cells were treated with or without CFF-1 incubated for 10 days at 37°C. Cells were fixed in 4% paraformaldehyde and stained with crystal violet. The numbers of visible colonies were counted under an optical microscope.

### Apoptosis assay

PC-3 cells were treated with various concentrations of CFF-1. After 24 h, apoptosis was detected by the annexin V-FITC apoptosis detection kit (cat. #640932, Biolegend, USA) adhering to the manufacturer’s protocol.

### Wound healing assay

PC-3 cells were added into 6-well plates and incubated until 100% confluence. After scraping in a straight line using a 200 μl pipette tip, the cells were rinsed thrice with PBS. Varying concentration of CFF-1 was administered to each well. The cell migration data were acquired with an inverted microscope Olympus IX51 at 0 and 24 h incubation and assessed using Image-Pro Plus 7.0 software.

### Transwell migration and invasion assay

The transwell migration and invasion assay was performed using a transwell chamber system with an 8-μm pore polycarbonate membrane (Thermo Fisher Scientific, Waltham, MA, USA). For migration assay, 1 × 10^5^ cells containing different concentrations of CFF-1 intervention and medium supplemented with 2% serum were plated onto 24-well chambers. For invasion assay, we diluted the Matrigel 1:4 with serum-free medium and seeded it to the upper chambers. Then, the cells were placed onto the upper chambers. Both assays were conducted as previously described [43].

### Western blot

Total protein was extracted using RIPA buffer containing proteinase inhibitor (Best Biological, Jiangsu, China). Western blot was conducted as previously described [12]. The primary antibodies for P-ERK1, ERK1, NF-κB1, RELA, P-mTOR, mTOR, VEGFA, PD-L1, P-P13K, P13K, P-AKT, AKT, TNF-α, P-EGFR, EGFR and HIF-1α were purchased from Bioss (Beijing, China). Total protein level was normalized to β-actin.

### ELISA

PC3 and CWR22Rv1 cells treated with different concentration of CFF-1 were cultured for 48 h. The supernatant of cell culture was collected and centrifuged at 1,000 g for 20 min. ELISA kits (Mlbio, Shanghai, China) were used to examine levels of IL-6 following the manufacture’s instruction. We measured the absorbance at 450 nm with a microplate reader.

### Statistical analysis

Statistical analyses of the *in vitro* PCa cell assays were conducted using SPSS 20.0 software. One-way univariate analysis of variance (ANOVA) was employed to analyze data obtained from at least three independent experiments, which are presented as the mean ± standard deviation. Statistical significance was defined as *p* < 0.05.

### Availability of data and materials

All data included in this study are available by contacting the corresponding authors.

## Supplementary Materials

Supplementary Figures

Supplementary Tables

## References

[1] Genkinger JM, Wu K, Wang M, Albanes D, Black A, van den Brandt PA, Burke KA, Cook MB, Gapstur SM, Giles GG, Giovannucci E, Goodman GG, Goodman PJ, et al. Measures of body fatness and height in early and mid-to-late adulthood and prostate cancer: risk and mortality in The Pooling Project of Prospective Studies of Diet and Cancer. Ann Oncol. 2020; 31:103–14. 10.1016/j.annonc.2019.09.007 31912782 PMC8195110

[2] Berg KD, Thomsen FB, Mikkelsen MK, Ingimarsdóttir IJ, Hansen RB, Kejs AM, Brasso K. Improved survival for patients with de novo metastatic prostate cancer in the last 20 years. Eur J Cancer. 2017; 72:20–7. 10.1016/j.ejca.2016.11.025 28024263

[3] Marra G, Valerio M, Heidegger I, Tsaur I, Mathieu R, Ceci F, Ploussard G, van den Bergh RCN, Kretschmer A, Thibault C, Ost P, Tilki D, Kasivisvanathan V, et al, and EAU-YAU Prostate Cancer Working Party. Management of Patients with Node-positive Prostate Cancer at Radical Prostatectomy and Pelvic Lymph Node Dissection: A Systematic Review. Eur Urol Oncol. 2020; 3:565–81. 10.1016/j.euo.2020.08.005 32933887

[4] Freedland SJ, Aronson WJ. Commentary on "Integrative clinical genomics of advanced prostate cancer". Robinson D, Van Allen EM, Wu YM, Schultz N, Lonigro RJ, Mosquera JM, Montgomery B, Taplin ME, Pritchard CC, Attard G, Beltran H, Abida W, Bradley RK, Vinson J, Cao X, Vats P, Kunju LP, Hussain M, Feng FY, Tomlins SA, Cooney KA, Smith DC, Brennan C, Siddiqui J, Mehra R, Chen Y, Rathkopf DE, Morris MJ, Solomon SB, Durack JC, Reuter VE, Gopalan A, Gao J, Loda M, Lis RT, Bowden M, Balk SP, Gaviola G, Sougnez C, Gupta M, Yu EY, Mostaghel EA, Cheng HH, Mulcahy H, True LD, Plymate SR, Dvinge H, Ferraldeschi R, Flohr P, Miranda S, Zafeiriou Z, Tunariu N, Mateo J, Perez-Lopez R, Demichelis F, Robinson BD, Schiffman M, Nanus DM, Tagawa ST, Sigaras A, Eng KW, Elemento O, Sboner A, Heath EI, Scher HI, Pienta KJ, Kantoff P, de Bono JS, Rubin MA, Nelson PS, Garraway LA, Sawyers CL, Chinnaiyan AM.Cell. 21 May 2015;161(5):1215-1228. Urol Oncol. 2017; 35:535. 10.1016/j.urolonc.2017.05.010 28623072

[5] Zhang D, Wang Z, Li J, Zhu J. Exploring the possible molecular targeting mechanism of Saussurea involucrata in the treatment of COVID-19 based on bioinformatics and network pharmacology. Comput Biol Med. 2022; 146:105549. 10.1016/j.compbiomed.2022.105549 35751193 PMC9035664

[6] Zhou X, Hong Y, Zhan Y. Karacoline, identified by network pharmacology, reduces degradation of the extracellular matrix in intervertebral disc degeneration via the NF-κB signaling pathway. J Pharm Anal. 2020; 10:13–22. 10.1016/j.jpha.2019.07.002 32123596 PMC7037626

[7] Xu HY, Zhang YQ, Liu ZM, Chen T, Lv CY, Tang SH, Zhang XB, Zhang W, Li ZY, Zhou RR, Yang HJ, Wang XJ, Huang LQ. ETCM: an encyclopaedia of traditional Chinese medicine. Nucleic Acids Res. 2019; 47:D976–82. 10.1093/nar/gky987 30365030 PMC6323948

[8] Li H, Liu Z, Liu L, Li W, Cao Z, Song Z, Yang Q, Lu A, Lu C, Liu Y. Vascular Protection of TPE-CA on Hyperhomocysteinemia-induced Vascular Endothelial Dysfunction through AA Metabolism Modulated CYPs Pathway. Int J Biol Sci. 2019; 15:2037–50. 10.7150/ijbs.35245 31592228 PMC6775291

[9] Wang N, Li P, Hu X, Yang K, Peng Y, Zhu Q, Zhang R, Gao Z, Xu H, Liu B, Chen J, Zhou X. Herb Target Prediction Based on Representation Learning of Symptom related Heterogeneous Network. Comput Struct Biotechnol J. 2019; 17:282–90. 10.1016/j.csbj.2019.02.002 30867892 PMC6396098

[10] Xu L, Liu Y, Wu H, Wu H, Liu X, Zhou A. Rapid identification of chemical profile in Gandou decoction by UPLC-Q-TOF-MS(E) coupled with novel informatics UNIFI platform. J Pharm Anal. 2020; 10:35–48. 10.1016/j.jpha.2019.05.003 32123598 PMC7037531

[11] Wu Z, Zhu Q, Yin Y, Kang D, Cao R, Tian Q, Zhang Y, Lu S, Liu P. Traditional Chinese Medicine CFF-1 induced cell growth inhibition, autophagy, and apoptosis via inhibiting EGFR-related pathways in prostate cancer. Cancer Med. 2018; 7:1546–59. 10.1002/cam4.1419 29533017 PMC5911605

[12] Zhang Y, Wei Y, Jiang S, Dang Y, Yang Y, Zuo W, Zhu Q, Liu P, Gao Y, Lu S. Traditional Chinese medicine CFF-1 exerts a potent anti-tumor immunity to hinder tumor growth and metastasis in prostate cancer through EGFR/JAK1/STAT3 pathway to inhibit PD-1/PD-L1 checkpoint signaling. Phytomedicine. 2022; 99:153939. 10.1016/j.phymed.2022.153939 35172257

[13] Huang S, Mu F, Li F, Wang W, Chen H, Lei L, Ma Y, Ding Y, Wang J. A Network-Based Approach to Explore the Mechanism and Bioactive Compounds of Erzhi Pill against Metabolic Dysfunction-Associated Fatty Liver Disease. J Diabetes Res. 2020; 2020:7867245. 10.1155/2020/7867245 32724826 PMC7382733

[14] Wu W, Zhang Z, Li F, Deng Y, Lei M, Long H, Hou J, Wu W. A Network-Based Approach to Explore the Mechanisms of *Uncaria* Alkaloids in Treating Hypertension and Alleviating Alzheimer's Disease. Int J Mol Sci. 2020; 21:1766. 10.3390/ijms21051766 32143538 PMC7084279

[15] Tortorella E, Giantulli S, Sciarra A, Silvestri I. AR and PI3K/AKT in Prostate Cancer: A Tale of Two Interconnected Pathways. Int J Mol Sci. 2023; 24:2046. 10.3390/ijms24032046 36768370 PMC9917224

[16] Hamidi A, Song J, Thakur N, Itoh S, Marcusson A, Bergh A, Heldin CH, Landström M. TGF-β promotes PI3K-AKT signaling and prostate cancer cell migration through the TRAF6-mediated ubiquitylation of p85α. Sci Signal. 2017; 10:eaal4186. 10.1126/scisignal.aal4186 28676490

[17] Hashemi M, Taheriazam A, Daneii P, Hassanpour A, Kakavand A, Rezaei S, Hejazi ES, Aboutalebi M, Gholamrezaie H, Saebfar H, Salimimoghadam S, Mirzaei S, Entezari M, Samarghandian S. Targeting PI3K/Akt signaling in prostate cancer therapy. J Cell Commun Signal. 2023; 17:423–43. 10.1007/s12079-022-00702-1 36367667 PMC10409967

[18] Li G, Kanagasabai T, Lu W, Zou MR, Zhang SM, Celada SI, Izban MG, Liu Q, Lu T, Ballard BR, Zhou X, Adunyah SE, Matusik RJ, et al. KDM5B Is Essential for the Hyperactivation of PI3K/AKT Signaling in Prostate Tumorigenesis. Cancer Res. 2020; 80:4633–43. 10.1158/0008-5472.CAN-20-0505 32868382 PMC8034842

[19] Won YS, Seo KI. Sanggenol L Induces Apoptosis and Cell Cycle Arrest via Activation of p53 and Suppression of PI3K/Akt/mTOR Signaling in Human Prostate Cancer Cells. Nutrients. 2020; 12:488. 10.3390/nu12020488 32075054 PMC7071324

[20] Yu J, Qi H, Wang Z, Zhang Z, Song E, Song W, An R. RAB3D, upregulated by aryl hydrocarbon receptor (AhR), promotes the progression of prostate cancer by activating the PI3K/AKT signaling pathway. Cell Biol Int. 2022; 46:2246–56. 10.1002/cbin.11910 36153645

[21] Atretkhany KN, Gogoleva VS, Drutskaya MS, Nedospasov SA. Distinct modes of TNF signaling through its two receptors in health and disease. J Leukoc Biol. 2020; 107:893–905. 10.1002/JLB.2MR0120-510R 32083339

[22] Kalliolias GD, Ivashkiv LB. TNF biology, pathogenic mechanisms and emerging therapeutic strategies. Nat Rev Rheumatol. 2016; 12:49–62. 10.1038/nrrheum.2015.169 26656660 PMC4809675

[23] Maolake A, Izumi K, Natsagdorj A, Iwamoto H, Kadomoto S, Makino T, Naito R, Shigehara K, Kadono Y, Hiratsuka K, Wufuer G, Nastiuk KL, Mizokami A. Tumor necrosis factor-α induces prostate cancer cell migration in lymphatic metastasis through CCR7 upregulation. Cancer Sci. 2018; 109:1524–31. 10.1111/cas.13586 29575464 PMC5980342

[24] Cowman SJ, Koh MY. Revisiting the HIF switch in the tumor and its immune microenvironment. Trends Cancer. 2022; 8:28–42. 10.1016/j.trecan.2021.10.004 34743924 PMC8702465

[25] Bao L, Chen Y, Lai HT, Wu SY, Wang JE, Hatanpaa KJ, Raisanen JM, Fontenot M, Lega B, Chiang CM, Semenza GL, Wang Y, Luo W. Methylation of hypoxia-inducible factor (HIF)-1α by G9a/GLP inhibits HIF-1 transcriptional activity and cell migration. Nucleic Acids Res. 2018; 46:6576–91. 10.1093/nar/gky449 29860315 PMC6061882

[26] Chen Y, Zhang B, Bao L, Jin L, Yang M, Peng Y, Kumar A, Wang JE, Wang C, Zou X, Xing C, Wang Y, Luo W. ZMYND8 acetylation mediates HIF-dependent breast cancer progression and metastasis. J Clin Invest. 2018; 128:1937–55. 10.1172/JCI95089 29629903 PMC5919820

[27] Sun X, Huang Q, Peng F, Wang J, Zhao W, Guo G. Expression and Clinical Significance of HKII and HIF-1α in Grade Groups of Prostate Cancer. Front Genet. 2021; 12:680928. 10.3389/fgene.2021.680928 34220956 PMC8248182

[28] Ucaryilmaz Metin C, Ozcan G. The HIF-1α as a Potent Inducer of the Hallmarks in Gastric Cancer. Cancers (Basel). 2022; 14:2711. 10.3390/cancers14112711 35681691 PMC9179860

[29] Huang M, Du H, Zhang L, Che H, Liang C. The association of HIF-1α expression with clinicopathological significance in prostate cancer: a meta-analysis. Cancer Manag Res. 2018; 10:2809–16. 10.2147/CMAR.S161762 30174456 PMC6109649

[30] Mazurakova A, Koklesova L, Csizmár SH, Samec M, Brockmueller A, Šudomová M, Biringer K, Kudela E, Pec M, Samuel SM, Kassayova M, Hassan STS, Smejkal K, et al. Significance of flavonoids targeting PI3K/Akt/HIF-1α signaling pathway in therapy-resistant cancer cells - A potential contribution to the predictive, preventive, and personalized medicine. J Adv Res. 2024; 55:103–18. 10.1016/j.jare.2023.02.015 36871616 PMC10770105

[31] Ouyang C, Zhang J, Lei X, Xie Z, Liu X, Li Y, Huang S, Wang Z, Tang G. Advances in antitumor research of HIF-1α inhibitor YC-1 and its derivatives. Bioorg Chem. 2023; 133:106400. 10.1016/j.bioorg.2023.106400 36739684

[32] Ozcan G. The hypoxia-inducible factor-1α in stemness and resistance to chemotherapy in gastric cancer: Future directions for therapeutic targeting. Front Cell Dev Biol. 2023; 11:1082057. 10.3389/fcell.2023.1082057 36846589 PMC9945545

[33] Rose-John S, Jenkins BJ, Garbers C, Moll JM, Scheller J. Targeting IL-6 trans-signalling: past, present and future prospects. Nat Rev Immunol. 2023; 23:666–81. 10.1038/s41577-023-00856-y 37069261 PMC10108826

[34] Culig Z, Puhr M. Interleukin-6 and prostate cancer: Current developments and unsolved questions. Mol Cell Endocrinol. 2018; 462:25–30. 10.1016/j.mce.2017.03.012 28315704

[35] Yamaguchi M, Hashimoto K, Jijiwa M, Murata T. The inflammatory macrophages repress the growth of bone metastatic human prostate cancer cells via TNF-α and IL-6 signaling: Involvement of cell signaling regulator regucalcin. Cell Signal. 2023; 107:110663. 10.1016/j.cellsig.2023.110663 37001596

[36] Yang F, Yuan C, Wu D, Zhang J, Zhou X. IRE1α Expedites the Progression of Castration-Resistant Prostate Cancers *via* the Positive Feedback Loop of IRE1α/IL-6/AR. Front Oncol. 2021; 11:671141. 10.3389/fonc.2021.671141 34295814 PMC8290131

[37] Xu Y, Song G, Xie S, Jiang W, Chen X, Chu M, Hu X, Wang ZW. The roles of PD-1/PD-L1 in the prognosis and immunotherapy of prostate cancer. Mol Ther. 2021; 29:1958–69. 10.1016/j.ymthe.2021.04.029 33932597 PMC8178461

[38] Gevensleben H, Dietrich D, Golletz C, Steiner S, Jung M, Thiesler T, Majores M, Stein J, Uhl B, Müller S, Ellinger J, Stephan C, Jung K, et al. The Immune Checkpoint Regulator PD-L1 Is Highly Expressed in Aggressive Primary Prostate Cancer. Clin Cancer Res. 2016; 22:1969–77. 10.1158/1078-0432.CCR-15-2042 26573597

[39] Shostak K, Chariot A. EGFR and NF-κB: partners in cancer. Trends Mol Med. 2015; 21:385–93. 10.1016/j.molmed.2015.04.001 25979753

[40] Liao Y, Guo Z, Xia X, Liu Y, Huang C, Jiang L, Wang X, Liu J, Huang H. Inhibition of EGFR signaling with Spautin-1 represents a novel therapeutics for prostate cancer. J Exp Clin Cancer Res. 2019; 38:157. 10.1186/s13046-019-1165-4 30975171 PMC6460657

[41] Newman AM, Liu CL, Green MR, Gentles AJ, Feng W, Xu Y, Hoang CD, Diehn M, Alizadeh AA. Robust enumeration of cell subsets from tissue expression profiles. Nat Methods. 2015; 12:453–7. 10.1038/nmeth.3337 25822800 PMC4739640

[42] Yoshihara K, Shahmoradgoli M, Martínez E, Vegesna R, Kim H, Torres-Garcia W, Treviño V, Shen H, Laird PW, Levine DA, Carter SL, Getz G, Stemke-Hale K, et al. Inferring tumour purity and stromal and immune cell admixture from expression data. Nat Commun. 2013; 4:2612. 10.1038/ncomms3612 24113773 PMC3826632

[43] Wei G, Yang X, Lu H, Zhang L, Wei Y, Li H, Zhu M, Zhou X. Prognostic value and immunological role of FOXM1 in human solid tumors. Aging (Albany NY). 2022; 14:9128–48. 10.18632/aging.204394 36435510 PMC9740373

