# Supplementary material for: Network pharmacology and experimental evaluation strategies to decipher the underlying pharmacological mechanism of Traditional Chinese Medicine CFF-1 against prostate cancer
Source: Aging (Albany NY). 2024 Mar 13;16(6):5387–411. doi: 10.18632/aging.205654 (PMC11006490; doi:10.18632/aging.205654)
Supplement: Supplementary Figures [file aging-16-205654-s001.pdf]

SUPPLEMENTARY FIGURES

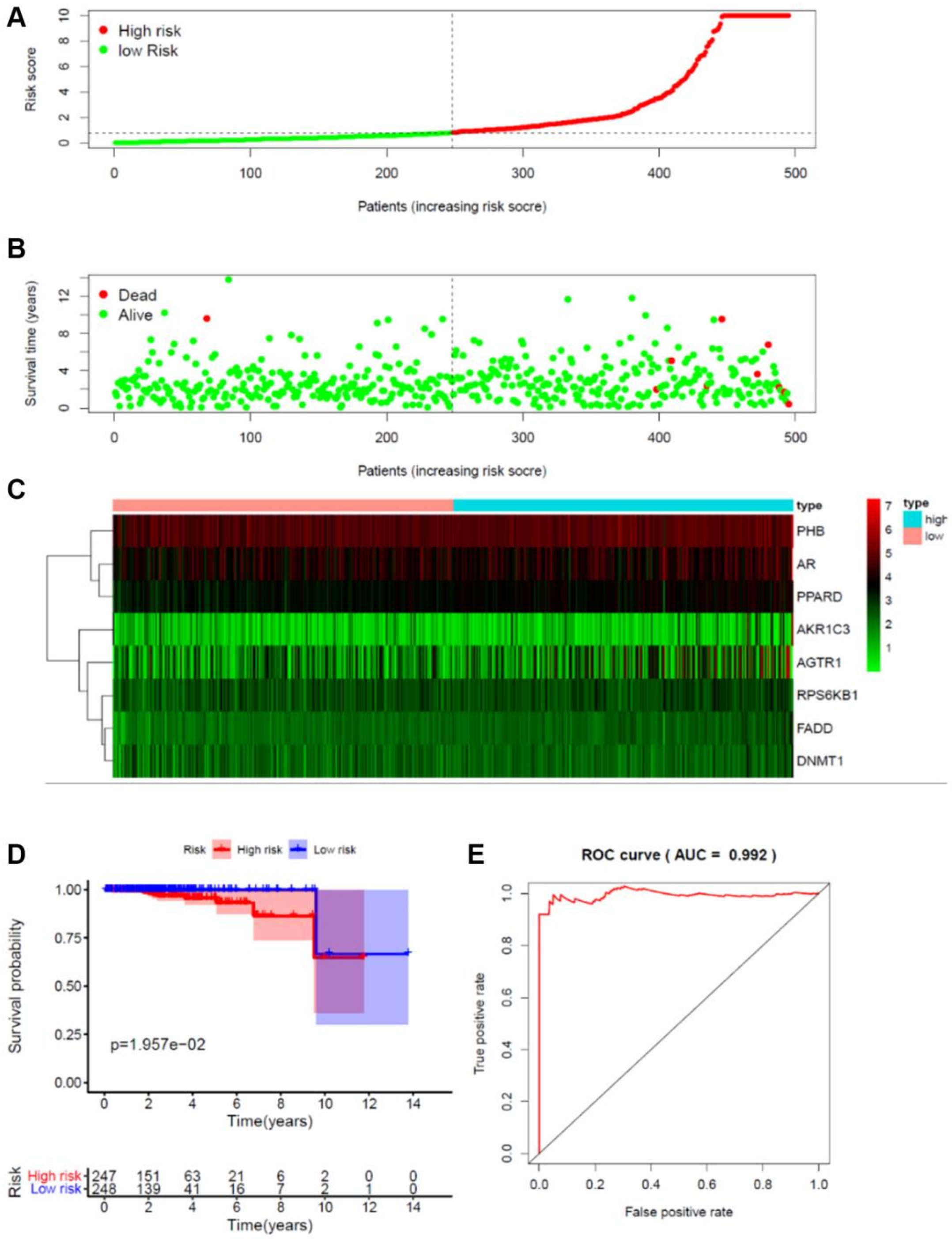

Supplementary Figure 1. Risk score for target gene signature and OS in PCa patients. (**A**) Risk score of an 8-gene signature for predicting OS. (**B**) Survival status and duration of cases. (**C**) Heatmap of the 8 gene expression in PCa patients. (**D**) Kaplan-Meier curve for OS in the low- and high-risk groups. (**E**) ROC curve for the OS prediction of risk score model.

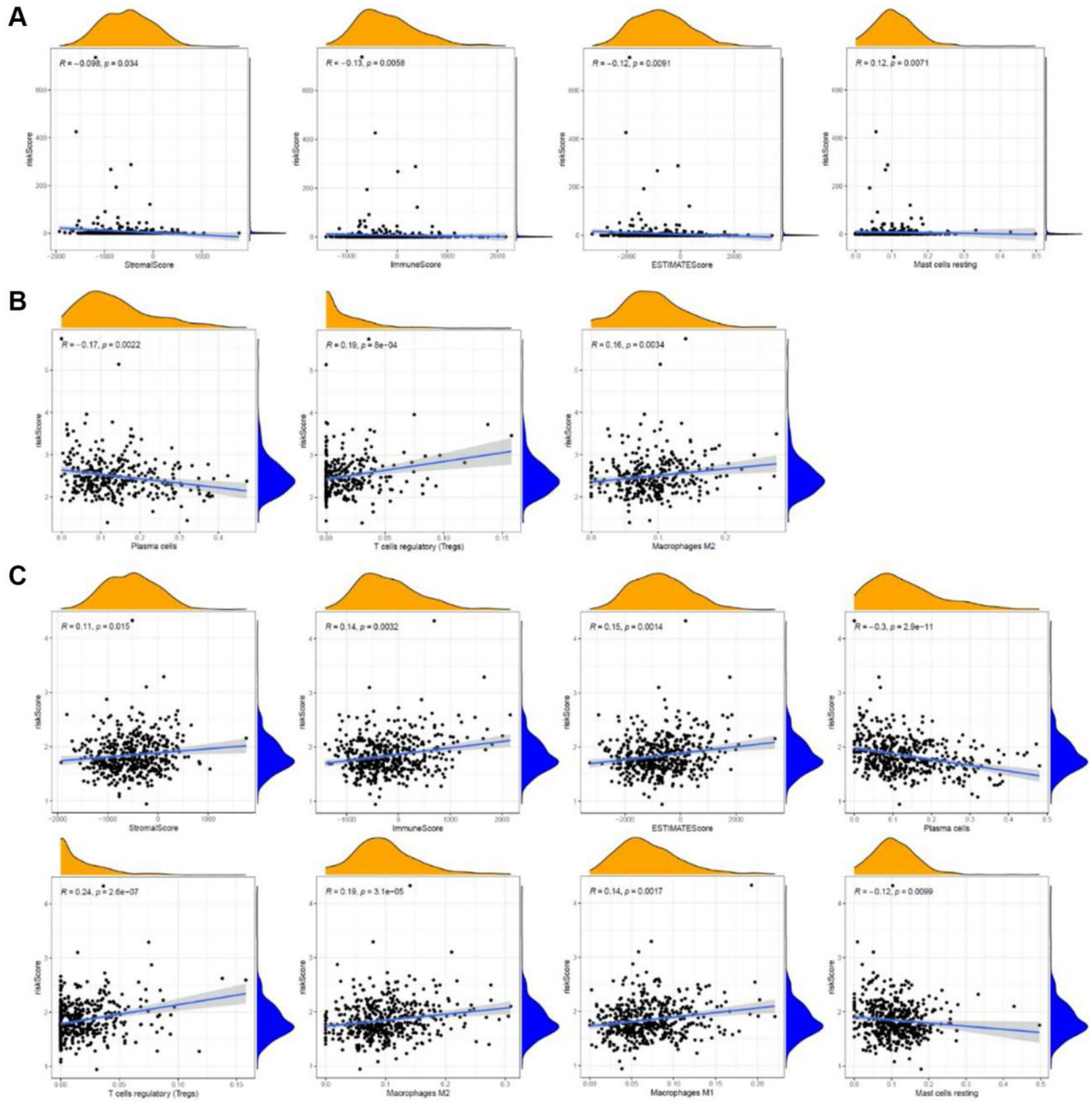

Supplementary Figure 2. The significant associations of the risk scores with immune score, stromal score, ESTIMATE score, and the infiltration of 22 immune cell subtypes. (**A**) The associations of risk score for predicting OS of PCa patients with immune score, stromal score, ESTIMATE score, and the infiltration of immune cell subtypes. (**B**) The associations of risk score for predicting DFS of PCa patients with the infiltration of immune cell subtypes. (**C**) The associations of risk score for predicting PFS of PCa patients with immune score, stromal score, ESTIMATE score, and the infiltration of 22 immune cell subtypes.

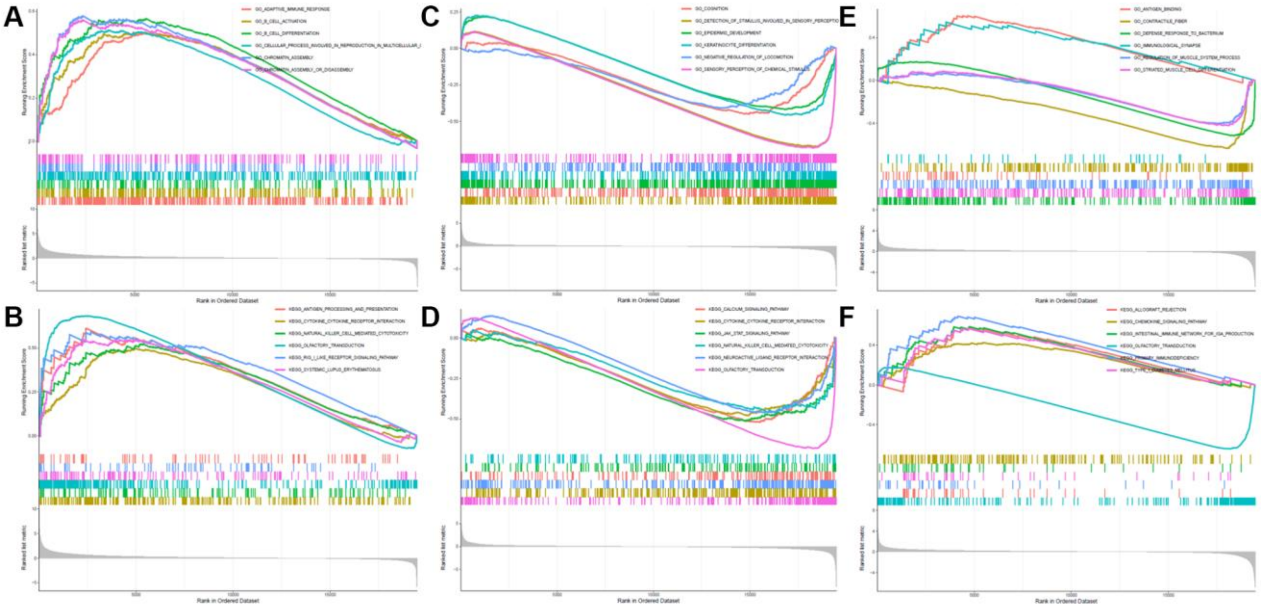

Supplementary Figure 3. (**A**, **B**) DNMT3B correlation with signaling pathways in GO (**A**) and KEGG (**B**) collection. (**C**, **D**) HPRT1 correlation with signaling pathways in GO (**C**) and KEGG (**D**) collection. (**E**, **F**) RXRB correlation with signaling pathways in GO (**E**) and KEGG (**F**) collection.
