# Supplementary material for: Network pharmacology and experimental evaluation strategies to decipher the underlying pharmacological mechanism of Traditional Chinese Medicine CFF-1 against prostate cancer
Source: Aging (Albany NY). 2024 Mar 13;16(6):5387–411. doi: 10.18632/aging.205654 (PMC11006490; doi:10.18632/aging.205654)
Supplement: Supplementary Tables [file aging-16-205654-s002.pdf]

SUPPLEMENTARY TABLES

Supplementary Table 1. KEGG pathway analysis of 359 key targets.

Pathway	*p*-value	Pathway	*p*-value	Pathway	*p*-value
Lipid and atherosclerosis	3.72E-25	Fluid shear stress and atherosclerosis	1.38E-22	Chagas disease	9.58E-22
AGE-RAGE signaling pathway in diabetic complications	6.00E-20	HIF-1 signaling pathway	8.91E-20	Prostate cancer	1.73E-19
Proteoglycans in cancer	8.59E-19	Hepatitis B	1.68E-18	Human cytomegalovirus infection	4.75E-18
Non-alcoholic fatty liver disease	5.33E-18	Thyroid hormone signaling pathway	1.71E-17	TNF signaling pathway	1.19E-16
Th17 cell differentiation	2.61E-16	Colorectal cancer	4.38E-16	EGFR tyrosine kinase inhibitor resistance	4.38E-16
IL-17 signaling pathway	4.61E-16	PI3K-Akt signaling pathway	8.65E-16	Gastric cancer	1.27E-15
Kaposi sarcoma-associated herpesvirus infection	3.27E-15	African trypanosomiasis	8.33E-15	Endocrine resistance	1.19E-14
Breast cancer	3.77E-14	Pancreatic cancer	1.68E-13	Hepatitis C	2.51E-13
Human immunodeficiency virus 1 infection	3.59E-13	Tuberculosis	3.67E-13	Toll-like receptor signaling pathway	4.16E-13
Influenza A	5.20E-13	Acute myeloid leukemia	8.91E-13	Toxoplasmosis	2.49E-12
PD-L1 expression and PD-1 checkpoint pathway in cancer	5.28E-12	MAPK signaling pathway	5.81E-12	Apoptosis	6.46E-12
Insulin resistance	6.90E-12	Osteoclast differentiation	8.63E-12	Measles	1.05E-11
Human papillomavirus infection	1.73E-11	Prolactin signaling pathway	1.88E-11	C-type lectin receptor signaling pathway	1.88E-11
Antifolate resistance	2.88E-11	Inflammatory bowel disease	4.00E-11	Platinum drug resistance	4.06E-11
Human T-cell leukemia virus 1 infection	7.71E-11	T cell receptor signaling pathway	1.24E-10	Parathyroid hormone synthesis, secretion and action	1.82E-10
Hepatocellular carcinoma	1.82E-10	Diabetic cardiomyopathy	1.96E-10	Yersinia infection	2.13E-10
Pathogenic Escherichia coli infection	4.06E-10	ErbB signaling pathway	7.10E-10	Leishmaniasis	8.45E-10
Adipocytokine signaling pathway	9.24E-10	Renal cell carcinoma	9.24E-10	Non-small cell lung cancer	1.95E-09
FoxO signaling pathway	2.33E-09	Small cell lung cancer	2.90E-09	Amoebiasis	3.03E-09
Epstein-Barr virus infection	3.03E-09	MicroRNAs in cancer	4.35E-09	Estrogen signaling pathway	6.48E-09
Neurotrophin signaling pathway	9.59E-09	Type II diabetes mellitus	9.61E-09	mTOR signaling pathway	1.41E-08
Alzheimer disease	1.63E-08	Shigellosis	2.14E-08	Endometrial cancer	2.91E-08
VEGF signaling pathway	3.70E-08	Relaxin signaling pathway	4.14E-08	AMPK signaling pathway	5.55E-08
Ras signaling pathway	7.09E-08	Th1 and Th2 cell differentiation	9.93E-08	NF-kappa B signaling pathway	1.29E-07
Focal adhesion	1.63E-07	Pertussis	1.93E-07	Chronic myeloid leukemia	1.93E-07
Sphingolipid signaling pathway	2.28E-07	Coronavirus disease - COVID-19	2.39E-07	Malaria	2.39E-07
Choline metabolism in cancer	2.47E-07	Fc epsilon RI signaling pathway	2.47E-07	Pathways of neurodegeneration - multiple diseases	2.97E-07
Longevity regulating pathway	3.04E-07	Salmonella infection	3.04E-07	Central carbon metabolism in cancer	3.54E-07
Aldosterone-regulated sodium reabsorption	5.05E-07	Thyroid cancer	5.05E-07	Melanoma	5.07E-07
B cell receptor signaling pathway	5.07E-07	Glioma	8.77E-07	Inflammatory mediator regulation of TRP channels	1.21E-06
Rap1 signaling pathway	1.22E-06	cAMP signaling pathway	2.10E-06	Chemokine signaling pathway	2.82E-06
Transcriptional misregulation in cancer	2.82E-06	Rheumatoid arthritis	2.84E-06	Longevity regulating pathway - multiple species	2.91E-06

Oxytocin signaling pathway	3.20E-06	TGF-beta signaling pathway	3.20E-06	Signaling pathways regulating pluripotency of stem cells	3.79E-06
Hippo signaling pathway	4.28E-06	Phospholipase D signaling pathway	6.43E-06	Insulin signaling pathway	7.72E-06
Apoptosis - multiple species	9.85E-06	Cellular senescence	1.42E-05	Basal cell carcinoma	2.02E-05
GnRH secretion	2.37E-05	Viral carcinogenesis	2.45E-05	JAK-STAT signaling pathway	2.45E-05
PPAR signaling pathway	2.80E-05	Platelet activation	2.82E-05	Cholinergic synapse	3.35E-05
NOD-like receptor signaling pathway	3.74E-05	Regulation of lipolysis in adipocytes	3.97E-05	Allograft rejection	4.16E-05
Cushing syndrome	4.27E-05	Natural killer cell mediated cytotoxicity	5.59E-05	Necroptosis	6.05E-05
Adherens junction	6.42E-05	Vascular smooth muscle contraction	6.65E-05	Bladder cancer	7.62E-05
Ovarian steroidogenesis	7.83E-05	Type I diabetes mellitus	0.000112	Progesterone-mediated oocyte maturation	0.00012
Regulation of actin cytoskeleton	0.000185	Legionellosis	0.000206	Neutrophil extracellular trap formation	0.000215
Growth hormone synthesis, secretion and action	0.000217	Calcium signaling pathway	0.000274	Prion disease	0.000288
Autophagy - animal	0.000313	Fc gamma R-mediated phagocytosis	0.000328	cGMP-PKG signaling pathway	0.000339
p53 signaling pathway	0.000362	Leukocyte transendothelial migration	0.000463	Melanogenesis	0.00048
Graft-versus-host disease	0.000534	Dopaminergic synapse	0.000646	GnRH signaling pathway	0.000796
Cytokine-cytokine receptor interaction	0.000886	Renin secretion	0.000936	RIG-I-like receptor signaling pathway	0.001046
Adrenergic signaling in cardiomyocytes	0.002414	Wnt signaling pathway	0.002467	Neuroactive ligand-receptor interaction	0.003181
Arginine biosynthesis	0.003267	Epithelial cell signaling in Helicobacter pylori infection	0.004089	Herpes simplex virus 1 infection	0.004216
Serotonergic synapse	0.004913	Viral myocarditis	0.005566	Gap junction	0.005838
Axon guidance	0.006027	Steroid hormone biosynthesis	0.006061	Intestinal immune network for IgA production	0.006578
Cocaine addiction	0.006578	Hypertrophic cardiomyopathy	0.006663	Cholesterol metabolism	0.007292
Autoimmune thyroid disease	0.010071	Retinol metabolism	0.011432	Amphetamine addiction	0.012406
Asthma	0.014127	Insulin secretion	0.014819	Tight junction	0.016568
Long-term depression	0.019059	Bile secretion	0.019386	Biosynthesis of amino acids	0.019535
Carbohydrate digestion and absorption	0.020249	Arachidonic acid metabolism	0.020299	Bacterial invasion of epithelial cells	0.022309
Cytosolic DNA-sensing pathway	0.023738	Tyrosine metabolism	0.024982	Oocyte meiosis	0.027646
Apelin signaling pathway	0.041067				

Supplementary Table 2. GO process analysis of 359 key targets.

Pathway	*p*-value	Pathway	*p*-value	Pathway	*p*-value
nuclear receptor activity	8.09E-21	transcription factor activity, direct ligand regulated sequence-specific DNA binding	8.09E-21	steroid hormone receptor activity	1.07E-20
receptor ligand activity	2.30E-20	receptor regulator activity	5.25E-20	cytokine receptor binding	2.70E-16
protein heterodimerization activity	9.69E-16	cofactor binding	1.06E-15	proximal promoter sequence-specific DNA binding	1.60E-14
RNA polymerase II proximal promoter sequence-specific DNA binding	1.60E-14	steroid binding	4.04E-14	chromatin binding	1.07E-13
cytokine activity	1.58E-13	heme binding	5.32E-11	hormone binding	5.33E-11
carboxylic acid binding	1.87E-10	phosphatase binding	1.98E-10	organic acid binding	2.09E-10
tetrapyrrole binding	2.09E-10	growth factor activity	2.30E-10	growth factor receptor binding	4.09E-10
hormone receptor binding	4.89E-10	coenzyme binding	1.01E-08	nuclear hormone receptor binding	1.14E-08
ubiquitin-like protein ligase binding	1.79E-08	oxidoreductase activity, acting on paired donors, with incorporation or reduction of molecular oxygen	2.35E-08	ubiquitin protein ligase binding	2.44E-08
vitamin binding	5.45E-08	monocarboxylic acid binding	8.89E-08	DNA-binding transcription activator activity, RNA polymerase II-specific	1.56E-07
NADP binding	1.56E-07	G protein-coupled receptor binding	4.22E-07	protein phosphatase binding	5.23E-07
tau protein binding	6.14E-07	transcription coregulator activity	6.14E-07	histone deacetylase binding	7.36E-07
protease binding	9.45E-07	nuclear receptor binding	9.59E-07	oxidoreductase activity, acting on the CH-CH group of donors	1.29E-06
phosphoprotein binding	1.29E-06	enzyme activator activity	2.02E-06	chemoattractant activity	2.02E-06
antioxidant activity	2.02E-06	estrogen receptor binding	2.70E-06	histone kinase activity	3.83E-06
monooxygenase activity	4.54E-06	amide binding	4.85E-06	steroid hormone receptor binding	5.53E-06
scaffold protein binding	5.53E-06	tumor necrosis factor receptor superfamily binding	5.53E-06	insulin-like growth factor receptor binding	5.53E-06
iron ion binding	6.67E-06	protein serine/threonine kinase activity	6.67E-06	E-box binding	1.11E-05
oxidoreductase activity, acting on the CH-CH group of donors, NAD or NADP as acceptor	1.14E-05	RNA polymerase II transcription factor binding	1.14E-05	integrin binding	1.14E-05
death receptor binding	1.14E-05	adrenergic receptor binding	1.14E-05	transcription coactivator activity	1.18E-05
kinase regulator activity	1.37E-05	protein serine/threonine/tyrosine kinase activity	2.01E-05	enhancer binding	2.28E-05
heat shock protein binding	3.90E-05	protein phosphorylated amino acid binding	4.11E-05	transcription coactivator binding	4.15E-05
oxidoreductase activity, acting on paired donors, with incorporation or reduction of molecular oxygen, NAD(P)H as one donor, and incorporation of one atom of oxygen	5.53E-05	electron transfer activity	7.64E-05	ammonium ion binding	7.64E-05
neurotransmitter binding	8.02E-05	protein tyrosine kinase activity	0.000105456	alcohol dehydrogenase (NADP+) activity	0.000108
NADPH binding	0.00010771	hormone activity	0.000125943	transcription cofactor binding	0.000133
oxygen binding	0.000176291	oxidoreductase activity, acting on the CH-OH group of donors, NAD or NADP as acceptor	0.00019025	fatty acid binding	0.000215
kinase activator activity	0.000254366	tumor necrosis factor receptor binding	0.000318127	insulin receptor substrate binding	0.000344
cholesterol transporter activity	0.00034585	disordered domain specific binding	0.00037338	insulin receptor binding	0.000442
oxidoreductase activity, acting on CH-OH group of donors	0.000460999	growth factor binding	0.000491722	activating transcription factor binding	0.000527
peptide binding	0.000532798	tau-protein kinase activity	0.00053822	oxidoreductase activity, acting on the aldehyde or oxo group of donors, NAD or NADP as acceptor	0.000596
dioxygenase activity	0.000661983	sterol transporter activity	0.000661983	flavin adenine dinucleotide binding	0.00068
sulfur compound binding	0.000680429	protein kinase activator activity	0.000748104	cysteine-type endopeptidase regulator activity involved in apoptotic process	0.00078
aldo-keto reductase (NADP) activity	0.000788194	phosphotyrosine residue binding	0.000895584	protein kinase A catalytic subunit binding	0.00091
retinoic acid receptor binding	0.000948814	Hsp90 protein binding	0.001013403	SMAD binding	0.001047
FAD binding	0.001131296	oxidoreductase activity, acting on the aldehyde or oxo group of donors	0.001142252	protein kinase regulator activity	0.001148
translation repressor activity, mRNA regulatory element binding	0.001148329	platelet-derived growth factor receptor binding	0.001148329	long-chain fatty acid binding	0.001148
protein self-association	0.001206353	oxidoreductase activity, acting on peroxide as acceptor	0.00124775	protein C-terminus binding	0.001257
retinoid X receptor binding	0.001484449	chromatin DNA binding	0.001513989	cholesterol binding	0.001578
cell adhesion molecule binding	0.001577986	repressing transcription factor binding	0.001577986	beta-catenin binding	0.001599

peptidase regulator activity	0.001614389	extracellular matrix binding	0.001735213	protein kinase C activity	0.001801
receptor serine/threonine kinase binding	0.001801096	NF-kappaB binding	0.001989279	heparin binding	0.002056
lipid transporter activity	0.002128739	nuclear receptor transcription coactivator activity	0.002128739	glycosaminoglycan binding	0.002137
modified amino acid binding	0.002634546	sterol binding	0.002643398	enhancer sequence-specific DNA binding	0.002716
transmembrane receptor protein tyrosine kinase activity	0.003269562	steroid hydroxylase activity	0.00336663	transforming growth factor beta receptor binding	0.004106
serine hydrolase activity	0.00427316	alcohol binding	0.00427316	fibroblast growth factor binding	0.00484
translation regulator activity, nucleic acid binding	0.004840122	peptidase activator activity	0.004971609	insulin-like growth factor I binding	0.00524
Toll-like receptor binding	0.005240093	NADP-retinol dehydrogenase activity	0.005240093	transmembrane receptor protein serine/threonine kinase binding	0.00524
RNA polymerase II activating transcription factor binding	0.005373752	peroxidase activity	0.005373752	non-membrane spanning protein tyrosine kinase activity	0.005374
mitogen-activated protein kinase binding	0.005455186	DNA-binding transcription repressor activity, RNA polymerase II-specific	0.005567454	oxidoreductase activity, acting on single donors with incorporation of molecular oxygen, incorporation of two atoms of oxygen	0.006361
translation repressor activity	0.006361033	folic acid binding	0.006577534	protein kinase B binding	0.006578
platelet-derived growth factor binding	0.006577534	I-SMAD binding	0.006577534	fibronectin binding	0.007213
oxidoreductase activity, acting on single donors with incorporation of molecular oxygen	0.007212618	transcription corepressor activity	0.007460244	catalytic activity, acting on DNA	0.007549
Hsp70 protein binding	0.007899398	interleukin-1 receptor binding	0.008250906	catecholamine binding	0.008251
voltage-gated cation channel activity	0.008952296	lipoprotein particle receptor binding	0.009383432	serine-type peptidase activity	0.009677
enzyme inhibitor activity	0.009974901	serine-type endopeptidase activity	0.009974901	MAP kinase kinase activity	0.010172
androgen receptor binding	0.010272915	G protein-coupled amine receptor activity	0.010318019	bHLH transcription factor binding	0.010318
protein serine/threonine kinase activator activity	0.010318019	neurotransmitter receptor activity	0.010348568	RNA polymerase II distal enhancer sequence-specific DNA binding	0.010897
transmembrane receptor protein kinase activity	0.01089714	protein kinase A binding	0.01089714	protein kinase C binding	0.011877
promoter-specific chromatin binding	0.011876902	retinol dehydrogenase activity	0.011945223	actinin binding	0.012818
protein phosphatase 2A binding	0.012817505	cation channel activity	0.013691175	translation regulator activity	0.013929
opsonin binding	0.014210904	lipase inhibitor activity	0.014210904	steroid dehydrogenase activity	0.015944
amyloid-beta binding	0.015944247	cysteine-type endopeptidase activator activity involved in apoptotic process	0.01666463	chloride channel regulator activity	0.016665
MHC class II protein complex binding	0.01666463	apolipoprotein binding	0.01666463	oxidoreductase activity, acting on NAD(P)H	0.017216
virus receptor activity	0.018832345	hijacked molecular function	0.018832345	ion gated channel activity	0.019364
DNA polymerase binding	0.019400305	gated channel activity	0.019649029	cysteine-type endopeptidase inhibitor activity	0.019649
copper ion binding	0.019649029	cytokine binding	0.02057876	lipoprotein particle binding	0.020579
protein-lipid complex binding	0.02057876	channel regulator activity	0.021625653	ATPase binding	0.021712
RNA polymerase II basal transcription factor binding	0.021784633	cyclin-dependent protein serine/threonine kinase regulator activity	0.021784633	retinoid binding	0.022287
channel activity	0.022469788	passive transmembrane transporter activity	0.022923969	cysteine-type endopeptidase activity	0.023506
isoprenoid binding	0.02412412	voltage-gated ion channel activity	0.02412412	voltage-gated channel activity	0.024124
peptidase activator activity involved in apoptotic process	0.024380968	cysteine-type endopeptidase inhibitor activity involved in apoptotic process	0.024380968	protein tyrosine kinase binding	0.024618
voltage-gated calcium channel activity	0.025914078	receptor tyrosine kinase binding	0.026221153	L-ascorbic acid binding	0.02771
protein N-terminus binding	0.029624628	RNA polymerase II core promoter sequence-specific DNA binding	0.031383956	neuropeptide hormone activity	0.031384
collagen binding	0.031583969	endopeptidase activity	0.032267629	oxidoreductase activity, acting on paired donors, with incorporation or reduction of molecular oxygen, 2-oxoglutarate as one donor, and incorporation of one atom each of oxygen into both donors	0.032873
lyase activity	0.03316175	double-stranded RNA binding	0.03316175	phospholipid binding	0.03808
calcium channel activity	0.03807999	core promoter binding	0.03807999	ion channel binding	0.038386
mRNA 5′-UTR binding	0.038386203	R-SMAD binding	0.038386203	fatty acid derivative binding	0.038386
p53 binding	0.038802999	chaperone binding	0.041073421	pyridoxal phosphate binding	0.043332
endopeptidase inhibitor activity	0.045706496	vitamin B6 binding	0.046377378	fibroblast growth factor receptor binding	0.046883
MHC protein complex binding	0.046883431				
